# Recent Advances in Nanotechnology-Based Approaches for Ferroptosis Therapy and Imaging Diagnosis in Pancreatic Cancer

**DOI:** 10.3390/pharmaceutics17070937

**Published:** 2025-07-20

**Authors:** Xiaoyan Yang, Wangping Luo, Yining Wang, Yongzhong Du, Risheng Yu

**Affiliations:** 1 Department of Radiology, The Second Affiliated Hospital, School of Medicine, Zhejiang University, 88 Jiefang Road, Hangzhou 310009, China; yangxiaoyandh@zju.edu.cn (X.Y.); wpluo@zju.edu.cn (W.L.); 22218732@zju.edu.cn (Y.W.); 2 Institute of Pharmaceutics, College of Pharmaceutical Sciences, Zhejiang University, 866 Yuhangtang Road, Hangzhou 310058, China

**Keywords:** nanotechnology, ferroptosis, imaging-guided therapy, theranostic, pancreatic cancer

## Abstract

Pancreatic cancer is a highly lethal malignant tumor characterized by challenges in early diagnosis and limited therapeutic options, leading to an exceptionally low clinical cure rate. With the advent of novel cancer treatment paradigms, ferroptosis—a form of iron-dependent regulated cell death driven by lipid peroxidation—has emerged as a promising therapeutic strategy, particularly for tumors harboring *RAS* mutations. However, the poor bioavailability and insufficient tumor-targeting capabilities of conventional drugs constrain the efficacy of ferroptosis-based therapies. Recent advancements in nanotechnology and imaging-guided treatments offer transformative solutions through targeted drug delivery, real-time monitoring of treatment efficacy, and multimodal synergistic strategies. This article aims to elucidate the mechanisms underlying ferroptosis in pancreatic cancer and to summarize the latest identified therapeutic targets for ferroptosis in this context. Furthermore, it reviews the recent progress in nanotechnology-based ferroptosis therapy for pancreatic cancer, encompassing ferroptosis monotherapy, synergistic ferroptosis therapy, and endogenous ferroptosis therapy. Subsequently, the integration of imaging-guided nanotechnology in ferroptosis therapy is summarized. Finally, this paper discusses innovative strategies, such as stroma-targeted ferroptosis therapy, immune-ferroptosis synergy, and AI-driven nanomedicine development, offering new insights and directions for future research in pancreatic cancer treatment.

## 1. Introduction

Pancreatic cancer is a highly aggressive malignancy with a 5-year survival rate of only 13%, and is expected to rise to be the second leading cause of cancer-related death in the United States by 2030 [1,2,3]. Due to the non-specific early symptoms, most of the pancreatic cancer cases are diagnosed at advanced stages. Although cancer treatments, such as surgery, chemotherapy, radiotherapy, targeted therapy and immunotherapy, have made significant advances in recent years, pancreatic cancer still shows strong resistance to these treatments [4]. Therefore, there is an urgent need to explore new therapeutic strategies to overcome these treatment challenges and to improve the prognosis of pancreatic cancer.

Programmed cell death has received extensive attention in the field of cancer therapy in recent years, such as apoptosis, necrosis, pyroptosis, and ferroptosis [5,6]. Ferroptosis is a unique non-apoptotic form of programmed cell death characterized by iron accumulation and lipid peroxide (LPO) production, leading to irreversible cell damage and death [7]. Studies have shown that tumor cells have an increased demand for iron, and are more susceptible to ferroptosis compared with normal cells [8,9,10]. Meanwhile, ferroptosis plays an important role in the regulation of the efficacy of chemotherapy, radiotherapy, and immunotherapy [11,12,13], and thus has emerged as a promising strategy for tackling cancers that are resistant to traditional therapies. Ferroptosis was originally defined as *RAS*-targeted therapy due to the fact that cancer cells with *RAS* mutations have shown a high sensitivity to ferroptosis induction by reprogramming cellular metabolism and compromising antioxidant defenses [14]. Given the high incidence of *RAS* mutations in pancreatic cancer, treatment strategies based on ferroptosis have considerable potential to improve treatment outcomes in pancreatic cancer.

**Figure 1:**
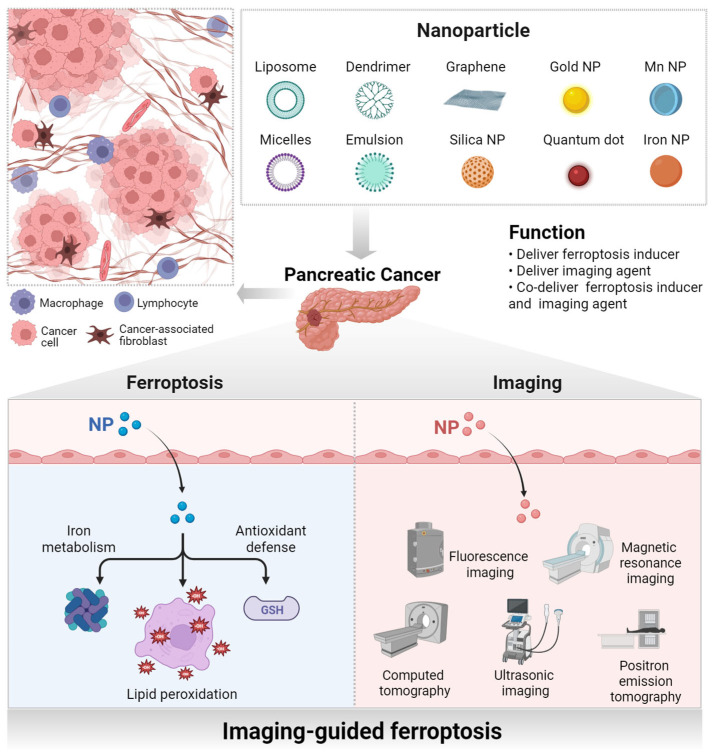
The application of nanotechnology in ferroptosis and imaging-guided therapy for pancreatic cancer. Created in BioRender. Xiaoyan, Y. (2025) https://BioRender.com/apmow4d. Abbreviations: NP: nanoparticle; GSH: glutathione.

Rapid advances in nanotechnology are providing new directions for cancer treatment, especially in targeted drug delivery and image-guided therapy. Nanomaterials can selectively induce ferroptosis in tumor cells by delivering exogenous iron or drugs that promote lipid peroxidation in tumor cells [15]. Utilizing the unique physical and chemical properties of nanomaterials, the combination of ferroptosis inducers and targeted nanocarriers can effectively enhance the bioavailability and tumor targeting of drugs. In addition, the inherent imaging properties of nanomaterials further broaden their application in tumor therapy. Image-guided therapy based on nanotechnology can precisely control drug release and monitor treatment progress in real time, further increasing the potential for the successful treatment of pancreatic cancer [16,17]. Therefore, combining ferroptosis with nanotechnology and image-guided therapy may open new avenues for the treatment of pancreatic cancer. Based on this, this article will introduce the treatment challenges of pancreatic cancer, as well as the mechanism of ferroptosis and its application in the treatment of pancreatic cancer. We will then discuss cutting-edge advances in nanotechnology and image-guided therapy in ferroptosis in pancreatic cancer, providing new strategies for the treatment dilemma of pancreatic cancer (Figure 1).

## 2. Treatment Challenges of Pancreatic Cancer

Pancreatic cancer is a highly heterogeneous and aggressive malignancy that requires the integration of multiple therapeutic strategies for treatment. The commonly used clinical treatments at present include surgery, chemotherapy, targeted therapy, and immunotherapy. Pancreaticoduodenectomy (Whipple procedure) is the most commonly performed surgery for pancreatic cancer, typically indicated for tumors located in the head of the pancreas [18], while distal pancreatectomy is preferred for cancers situated in the body or tail of the pancreas [19]. The feasibility of surgical treatment depends on the stage of cancer and the physical condition of patients. Since pancreatic cancer typically does not present noticeable symptoms in its early stages, most patients are diagnosed at an advanced stage. As a result, only about 15–20% of pancreatic cancer patients are eligible for surgical treatment [20]. Additionally, although surgery can remove visible tumors, the risk of recurrence remains high due to the aggressive and metastatic nature of the disease. Overall, the postoperative recurrence rate is typically between 70 and 80% [21,22]. Therefore, the majority of pancreatic cancer patients are not candidates for surgery, and treatment options will focus on non-surgical methods, such as chemotherapy. FOLFIRINOX (a combination of 5-fluorouracil (5-FU), leucovorin, irinotecan, and oxaliplatin) and gemcitabine in combination with nab-paclitaxel are currently recognized as the first-line chemotherapy regimens for pancreatic cancer; however, these treatments extend overall survival by only a few months [4,23,24]. Pancreatic cancer cells often demonstrate resistance to conventional chemotherapy agents, which can be attributed in part to the dense fibrosis within the tumor microenvironment that hinders drug penetration. The tumor microenvironment of pancreatic cancer is characterized by an abundance of fibrous tissue, known as “desmoplastic stroma”. This stromal component increases the rigidity and density of the tumor, thereby impeding drug diffusion, and consequently diminishing treatment efficacy [25,26,27]. The fibrotic response in pancreatic cancer is driven by excessive cancer-associated fibroblasts (CAFs) and an excessive extracellular matrix (ECM) deposition, which occupies the majority of the tumor mass [28]. CAFs represent the predominant stromal cell population in the pancreatic cancer tumor microenvironment, and serve as the primary producers and remodelers of the ECM. Pancreatic cancer cells induce a pro-fibrotic response within the tumor stroma by stimulating stromal CAFs to upregulate the expression of collagen family proteins and fibronectin in a paracrine manner [28,29]. In addition, tumor-associated macrophages (TAMs) are predominantly polarized toward an M2-like phenotype that promotes tumor progression. While they play a crucial but more regulatory role in ECM formation and remodeling, TAMs contribute to this process through multiple mechanisms: On one hand, they secrete potent CAF-activating factors (such as TGF-β and PDGF) to recruit and activate CAFs [30,31]; on the other hand, TAMs produce pro-angiogenic factors (such as VEGF, FGF2, and IL-8), and the resulting neovascularization not only supplies nutrients to the tumor but sustains continuous ECM synthesis and remodeling [32,33].

Compared to traditional chemotherapy, targeted therapy provides greater specificity and is associated with fewer side effects. However, due to the complex molecular landscape of pancreatic cancer, the effectiveness of targeted therapy remains limited. Similar to other solid tumors, the progression of pancreatic cancer is driven by the progressive accumulation of mutations in key driver genes, including the oncogene *KRAS* [34,35,36], the tumor suppressor gene *TP53* [37,38], cyclin-dependent kinase inhibitor 2A (*CDKN2A*), and SMAD family member 4 (*SMAD4*) [39,40,41]. Among them, *KRAS* mutations are the most common gene mutations in pancreatic cancer, occurring in approximately 90% of patients. Although many new molecules that directly or indirectly target *KRAS* have been developed in recent years, the resistance mechanism of *KRAS* is a major obstacle to targeted therapy [3,42]. Tumors often acquire secondary mutations or activate alternative signaling pathways to bypass *KRAS* inhibition, ultimately leading to the failure of targeted therapies [42,43]. Furthermore, *KRAS* mutations are intricately linked to tumor cell proliferation, survival, and immune evasion, thereby contributing to resistance against multiple therapeutic approaches [25,44,45].

Immunotherapy is an emerging treatment strategy in recent years that activates or boosts the immune system to recognize and attack cancer cells. While significant progress has been made in the treatment of certain cancers with immunotherapy in recent years, its application to pancreatic cancer remains a major challenge. The unique tumor microenvironment of pancreatic cancer and the complex immune escape mechanism of cancer cells greatly reduce the effectiveness of immunotherapy. Specifically, the tumor microenvironment of pancreatic cancer is usually rich in immunosuppressive cells, such as tumor-associated macrophages, regulatory T cells, and myeloid suppressor cells, which secrete immunosuppressive factors that inhibit anti-tumor immune responses [46,47,48]. At the same time, pancreatic cancer cells can also inhibit T cell activity by expressing PD-L1 and other immune checkpoint molecules [49,50]. In addition, the highly fibrotic extracellular matrix of pancreatic cancer not only limits the infiltration of immune cells but impedes the effective delivery of immunotherapeutic agents. Moreover, autophagy facilitates the immune escape of pancreatic cancer cells through the selective degradation of MHC Class I (MHC-I) proteins [51]. These immune evasion mechanisms make immunotherapies (such as immune checkpoint inhibitors) less effective in treating pancreatic cancer. Even though the immune checkpoint inhibitor, anti-PD-1 antibody, is currently the only approved therapy for solid tumors characterized by microsatellite instability-high (MSI-H), mismatch repair deficiency (dMMR), or high tumor mutational burden (TMB-high), these conditions are exceedingly uncommon in pancreatic cancer patients, occurring in merely 0–1.3% of the cases [42,52,53].

In addition to the aforementioned therapeutic challenges, early diagnosis of pancreatic cancer poses significant difficulties, primarily attributed to the insidious nature and non-specificity of its initial symptoms. Traditional imaging modalities, such as MRI and CT scans, frequently encounter challenges in detecting early-stage tumors, particularly when the lesions are small or situated deep within the pancreas [54]. Research on biomarkers, such as circulating tumor cells (CTCs) [55], circulating tumor DNA (ctDNA) [56,57], microRNAs (miRNAs) [58] and exosomes [59], is still in the exploratory phase, and current screening tools have not been able to effectively detect early-stage pancreatic cancer. Therefore, exploring new therapeutic modalities to overcome the treatment dilemma of pancreatic cancer remains the focus of current medical research.

## 3. Mechanisms of Ferroptosis and Emerging Therapeutic Targets in Pancreatic Cancer

**Figure 2:**
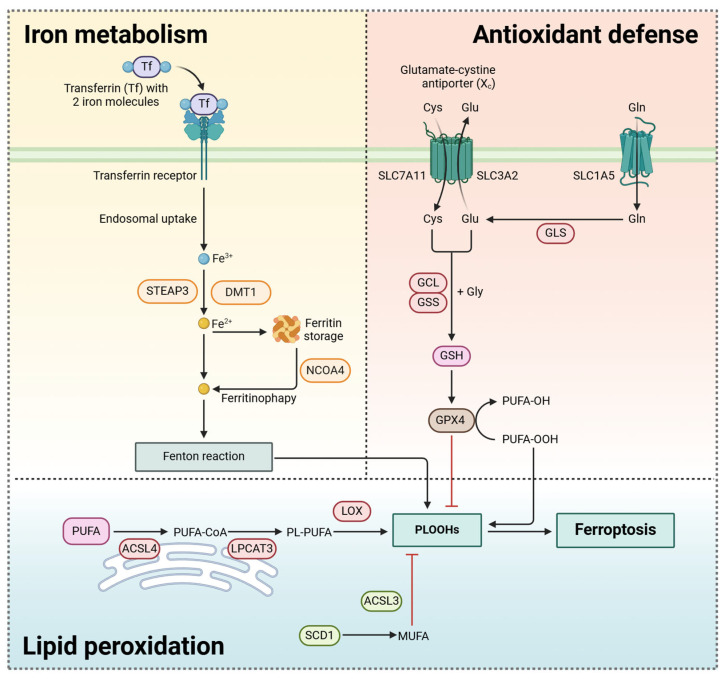
The mechanism of ferroptosis. Arrows represent activation processes, while perpendicular bars indicate inhibitory effects. Created in BioRender. Xiaoyan, Y. (2025) https://BioRender.com/qubgivz. Abbreviations: Tf: transferrin; STEAP3: six-transmembrane epithelial antigen of the prostate 3; DMT1: divalent metal transporter 1; NCOA4: nuclear receptor coactivator 4; Cys: cysteine; Glu: glutamate; Gln: glutamine; SLC7A11: solute carrier family 7 member 11; SLC3A2: solute carrier family 3 member 2; SLC1A5: solute carrier family 1 member 5; GLS: glutaminase; GCL: gamma-glutamylcysteine reductase; GSS: glutathione synthetase; GSH: glutathione; Gly: glycine; GPX4: glutathione peroxidase 4; PUFA-OH: polyunsaturated fatty acid hydroxyl group; PUFA-OOH: polyunsaturated fatty acid hydroperoxide; PUFA: polyunsaturated fatty acid; ACSL4: acyl-CoA synthetase long-chain family member 4; LPCAT3: lysophosphatidylcholine acyltransferase 3; PL-PUFA: polyunsaturated fatty acid–containing phospholipids; LOX: lipoxygenase; PLOOHs: phospholipid hydroperoxides; ACSL3: acyl-CoA synthetase long chain family member 3; SCD1: stearoyl-CoA desaturase 1; MUFA: monounsaturated Fatty Acid.

The concept of “ferroptosis” was first proposed by Scott J Dixon et al. in 2012 to describe a type of iron-dependent programmed cell death observed in *RAS*-mutated cancer cells [7]. Its primary characteristic is the excessive accumulation of iron ions and the subsequent lipid peroxidation, which disrupts the integrity of the cell membrane and ultimately leads to cell death. Unlike traditional apoptosis or necrosis, the process of ferroptosis involves intracellular iron metabolism, the production of lipid peroxides (LPOs), and the regulation of the antioxidant defense system [60]. Therefore, this section aims to provide a comprehensive review of the mechanisms of ferroptosis in pancreatic cancer (Figure 2). In addition, it will summarize the cutting-edge research on newly developed drugs or compounds targeting ferroptosis in pancreatic cancer.

### 3.1. Iron Metabolism

The iron metabolism process is one of the core mechanisms of ferroptosis, involving the accumulation, metabolism, and catalytic role of iron ions in lipid peroxidation. Iron acts as a key catalytic factor, promoting oxidation reactions, especially lipid peroxidation, which ultimately leads to cell death. The regulation of iron uptake, storage, and utilization is tightly controlled by multiple proteins and pathways, and dysregulation of these mechanisms can lead to iron overload and subsequent ferroptosis. First, two ferric (Fe^3+^) ions bind to transferrin (Tf) in the blood and enter the cell by binding to the transferrin receptor (TfR) on the cell membrane [61]. After entering the cell, the Tf-2Fe-TfR complex is encapsulated in the endosome by endocytosis. The acidic, low-pH environment of the endosome induces conformational changes in Tf and TfR, leading to the dissociation of iron from Tf [62]. After dissociation, the iron-free transferrin (apo-Tf) remains tightly bound to its receptor until it reaches the cell surface, where apo-Tf is recycled back into the extracellular fluid to further sequester free iron ions [63]. The released Fe^3+^ is reduced to ferrous iron (Fe^2+^) by the six-transmembrane epithelial antigen of the prostate 3 (STEAP3) [64]. The Fe^2+^ is then exported from the endosome into the cytoplasm via the divalent metal transporter 1 (DMT1) [61,65]. As one of the essential trace element nutrients, part of the endogenous iron is stored in the ferritin molecules of cells, while the remaining iron is distributed to various organelles within the cells to participate in functional activities. Functional iron in various enzymes, such as nicotinamide adenine dinucleotide (NADH) dehydrogenase, iron–sulfur protein, and cytochrome oxidase, exists in the organelles, including mitochondria, lysosomes, and the Golgi apparatus [66]. Ferritin stores Fe^2+^ in a non-toxic form, preventing excess iron from triggering oxidative stress and cell damage [7,67]. However, when cells require more iron during ferroptosis, stored iron is mobilized, increasing the concentration of free iron within the cell [68,69]. Free Fe^2+^ can participate in the Fenton reaction, generating highly reactive hydroxyl radicals (·OH), which is a core mechanism of ferroptosis. The generated ·OH directly attacks the lipids in the cell membrane, triggering lipid peroxidation and initiating ferroptosis.

Fenton Reaction: Fe^2+^ + H_2_O_2_ → Fe^3+^ + OH^−^ + ·OH

The regulation of iron metabolism involves several molecules that control iron absorption, storage, and release. Key iron regulatory factors include iron response proteins (IRPs), ferritin, and TfR. IRPs regulate iron absorption and storage by binding to iron-responsive elements (IREs) [68,69]. When intracellular iron concentrations are elevated, IRPs modulate the synthesis of ferritin and consequently decrease iron uptake. The expression of ferritin is regulated in response to intracellular iron levels. As intracellular iron accumulates, ferritin synthesis is upregulated to mitigate the accumulation of free iron [61,67]. In addition, TfR plays a crucial role in regulating cellular iron homeostasis by mediating iron uptake. Intracellular iron levels exert negative feedback regulation on TfR expression; specifically, iron deficiency within the cell leads to increased TfR expression, while iron excess results in decreased TfR expression [70]. Ferritinophagy is a specialized autophagic process in which ferritin is selectively engulfed into autophagosomes and subsequently degraded through the autophagic pathway. This process relies on nuclear receptor coactivator 4 (NCOA4), which plays a bridging role in transporting ferritin to the autophagosome [71]. Ferritinophagy plays a crucial role in cellular iron homeostasis and iron metabolism, especially when the demand for iron increases [72,73].

### 3.2. Generation of Lipid Peroxides (LPOs)

Lipid peroxidation is one of the hallmark events of ferroptosis, especially the lipid peroxidation of polyunsaturated fatty acids (PUFAs), which is a significant component of the lipid bilayer of cell membranes. The presence of multiple double bonds renders them particularly vulnerable to oxidative damage by free radicals. During ferroptosis, ·OH generated via the Fenton reaction catalyzed by iron specifically targets the PUFAs within the cell membrane, leading to the formation of LPOs, which not only damage the membrane structure but activate lipid peroxidation enzymes (such as lipoxygenases, LOXs), further amplifying the lipid peroxidation chain reaction [7,74]. The 5-LOX and 12-LOX enzymes are the key enzymes that catalyze lipid peroxidation in ferroptosis, promoting lipid peroxidation by inserting peroxide groups into the double bonds of PUFAs [75,76,77,78]. Acyl-CoA synthetase long-chain family member 4 (ACSL4) and lysophosphatidylcholine acyltransferase 3 (LPCAT3) in the endoplasmic reticulum are crucial for generating PUFA derivatives [79,80]. These derivatives undergo LOX-catalyzed lipid peroxidation, which produces toxic phospholipid hydroperoxides (PLOOHs) that trigger ferroptosis. On the other hand, ACSL3 and stearoyl-CoA desaturase 1 (SCD1) generate monounsaturated fatty acids (MUFAs) that can inhibit PUFA-induced ferroptosis by competing for incorporation into cell membranes [81,82]. MUFAs provide a protective mechanism against the occurrence of lipid peroxidation and ferroptosis, which highlights the heterogeneity of different lipids in the regulation of ferroptosis. Lipid synthesis and metabolism also have a significant effect on ferroptosis, which affects the composition of membrane lipids and their antioxidant capacity. The synthesis and accumulation of PUFAs in cell membranes provides the substrate for lipid peroxidation [74]. Therefore, altering the lipid biosynthesis pathway, such as the inhibition of fatty acid synthase (FASN), can reduce PUFAs synthesis, thereby reducing lipid peroxidation and ferroptosis [74,83,84]. Fatty acid oxidation is also closely related to ferroptosis. Beta-oxidation is an important metabolic pathway of fatty acids, and studies have shown that fatty acid oxidation products, such as lipid aldehydes, can exacerbate lipid peroxidation [7,74].

### 3.3. Antioxidant Defenses

Ferroptosis is usually accompanied by inhibition of intracellular antioxidant enzymes, resulting in a decrease in the cell’s ability to clear peroxide products, thus accelerating the cell death process. Glutathione peroxidase 4 (GPX4) is one of the key antioxidant enzymes in ferroptosis, which can inhibit ferroptosis by reducing the production of LPOs [85]. Specifically, GPX4 is a glutathione (GSH) dependent enzyme that converts PLOOHs into non-toxic phosphatidyl alcohols (PLOHs) within cell membranes, thereby preventing the further development of lipid peroxidation [60,85]. Since the activity of GPX4 depends on GSH, the consumption of GSH will lead to a decrease in the activity of GPX4.

The system Xc-, also known as the antiporter of cystine and glutamate, is a transmembrane transport system responsible for the uptake of cystine from the extracellular environment into the cell while exporting glutamate in the opposite direction. In this process, cystine is reduced to cysteine, and is subsequently involved in GSH biosynthesis, thereby maintaining cellular REDOX homeostasis [86,87]. The system Xc- is composed of solute carrier family 7 member 11 (SLC7A11) and solute carrier family 3 member 2 (SLC3A2), which work together to facilitate the import of cystine into cells [88]. Since the dysfunction of system Xc- can lead to cysteine deficiency, thereby reducing the synthesis of GSH and affecting the activity of GPX4, system Xc- plays an indirect regulatory role in ferroptosis. Classic ferroptosis inducers, such as erastin, promote ferroptosis by interfering with the function of system Xc- and reducing the activity of GPX4. By contrast, another ferroptosis inducer, RSL3, inhibits the activity of GPX4 by directly binding to it [7,89]. In addition to system Xc-, GSH synthesis is another key process involving the catalytic activity of the glutamate–cysteine ligase (GCL) and glutathione synthase (GSS). Therefore, these enzymes are also necessary for cells to maintain REDOX homeostasis.

### 3.4. Emerging Therapeutic Targets of Ferroptosis in Pancreatic Cancer

To date, only a few specific ferroptosis inducers, such as imidazole ketone erastin and sorafenib, have been demonstrated as effective anticancer agents in animal studies [90,91]. Therefore, we summarize the recent studies targeting potential ferroptosis-related pathways in pancreatic cancer, offering new insights for the development of ferroptosis-based therapeutic strategies in this malignancy. We will introduce these drugs or targets from the perspectives of regulating iron metabolism, lipid metabolism, and the antioxidant defense system.

#### 3.4.1. Targets or Drugs That Regulate Iron Metabolism

Lysosome plays a crucial role as a regulator of iron homeostasis within cells [92], and its acidic environment, in conjunction with the presence of bioavailable iron and H_2_O_2_, creates an optimal chemical milieu for the oxidation of membrane phospholipids. Based on this, a recent study developed a small-molecule activator of lysosomal iron (Fento-1) that leverages the elevated iron load in specific subpopulations of cancer cells to induce ferroptosis by activating lysosomal iron release [93]. Fento-1 demonstrates the ability to eliminate iron-rich pancreatic cancer cells characterized by high levels of CD44. In both primary pancreatic cancer cells and human pancreatic cancer-derived organoids, Fento-1 exhibits superior efficacy in reducing cell viability compared to standard therapeutic agents. Additionally, it acts synergistically with these conventional drugs in several primary human pancreatic cancer cell lines. These results indicate that controlling iron reactivity can control membrane lipid oxidation and establish lysosomal iron as a drug target, providing a reference for the design of ferroptosis regulators.

Tumor cells are capable of utilizing epithelial–mesenchymal plasticity to achieve a state of drug tolerance that is independent of genetic alterations [94,95]. Research has demonstrated that salinomycin can eliminate tumor cells in the mesenchymal state by sequestering iron within lysosomes, exploiting the iron dependency of this cellular condition. A recent study introduced a series of structurally intricate small-molecule chimeras comprising salinomycin derivatives and iron-reactive dihydroartemisinin (DHA) [96]. The researchers observed that these chimeric compounds accumulate in lysosomes and react with iron to release bioactive molecules, thereby inducing ferroptosis in drug-resistant pancreatic cancer cells and organoids. As ferroptosis represents a promising strategy to circumvent cancer drug resistance, this study provides an exciting and innovative approach for targeting persistently drug-resistant pancreatic cancer cells.

Autophagy-related genes (Atg) are essential for the process of autophagy in pancreatic cancer cells. When *Atg5* and *Atg7* are silenced, a reduction in Fe^2+^ levels and a decrease in the production of lipid peroxidation products are observed, which suggests that autophagy mediated by *Atg5* and *Atg7* is crucial for the induction of ferroptosis [72].

Research has shown that mitochondrial DNA (mtDNA) damage triggers an autophagic response within pancreatic cancer cells, which in turn facilitates the occurrence of ferroptosis. The study found that the antiviral drug zalcitabine induces mtDNA stress, activates the STING1/TMEM173-mediated DNA sensing pathway, and suppresses the growth of pancreatic cancer cells by inducing autophagy-dependent ferroptosis [97].

Myoferlin is an emerging oncoprotein that regulates mitochondrial structure and respiratory function, and is associated with low survival rates in various cancers. Researchers have found that targeting myoferlin with a compound WJ460 can induce mitophagy and activate ferroptosis in pancreatic cancer cells [98]. This research highlights myoferlin as a potential therapeutic target that may offer a novel strategy for the treatment of pancreatic cancer.

Selenium is an essential trace element in living organisms. To date, 25 selenium proteins have been identified in humans, many of which play a key role in REDOX homeostasis due to the unique biochemical properties of their selenium-containing portion [99]. Research has demonstrated that organoselenium compounds, such as diphenyl diselenide (DBDS), can induce ferroptosis in pancreatic cancer cells by impairing mitochondrial structure and function, also liberating organelle-buffered iron, thereby exacerbating intracellular oxidative stress [100]. The study also explored the combined effect of DBDS and *statins*. The mevalonate pathway (MVP) reduces oxidative stress by producing some radical-trapping isoprenoids. Simvastatin treatment decreased the abundance of MVP-derived CoQ in pancreatic cancer cells and promoted the production of ROS. The results demonstrated that co-treatment with DBDS and simvastatin exerted a synergistic effect, enhancing the induction of ferroptosis, thereby further inhibiting the proliferation of pancreatic cancer cells and promoting cell death [100].

#### 3.4.2. Targets or Drugs That Regulate Lipid Metabolism

The expression of transmembrane protein 164 (TMEM164) is closely associated with autophagic activity. By promoting autophagy, TMEM164 enhances the sensitivity of cells to ferroptosis. Studies have shown that inhibiting the expression or function of TMEM164 reduces the occurrence of ferroptosis, highlighting the critical role of TMEM164 in autophagy-dependent ferroptosis. TMEM164 influences lipid metabolism and the autophagic process, indirectly promoting the accumulation of LPOs, which in turn activates ferroptosis. This finding not only deepens our understanding of the relationship between ferroptosis and autophagy but provides a theoretical basis for developing new therapeutic strategies [101].

CAFs regulate lipid metabolism in pancreatic cancer cells by secreting exosomes containing miRNAs (miR-3173-5p) that target ACSL4, thereby inhibiting ACSL4 expression and reducing ferroptosis. Furthermore, these miRNAs enhance cancer cell resistance to gemcitabine by modulating lipid peroxidation [102]. This finding underscores the significant role of CAFs in the tumor microenvironment, particularly their ability to regulate ferroptosis and drug resistance in tumor cells through the exosomal transfer of miRNAs, offering new perspectives for the treatment of pancreatic cancer by targeting and inhibiting the functions of CAFs.

#### 3.4.3. Targets or Drugs That Regulate Antioxidant Systems

A recent study demonstrated that the knockout of the transcription factor AT-rich interactive domain-containing protein 3A (ARID3A) can inhibit tumor progression in pancreatic cancer and enhance the sensitivity of cells to gemcitabine. Further mechanistic investigations revealed that the knockout of ARID3A alleviates transcriptional repression of PTEN, which is a known tumor suppressor. PTEN inhibits the PI3K/AKT signaling pathway and plays a role in regulating the cellular antioxidant defense system. The upregulation of PTEN results in the reduced expression of GPX4, which leads to increased lipid peroxidation levels and subsequently activates ferroptosis. The study uncovers the important role of ARID3A in pancreatic cancer, particularly in enhancing chemoresistance by inhibiting PTEN-induced ferroptosis. Thus, ARID3A inhibition to promote PTEN-mediated ferroptosis offers a new approach for pancreatic cancer chemoresistance [103].

Thiostrepton (TST) is a protein translation inhibitor against Gram-positive and some Gram-negative bacteria, and has been shown to inhibit the malignant behaviors of various cancers. TST induces ferroptosis in pancreatic cancer cells by inhibiting GPX4 expression through the STAT3/GPX4 signaling pathway, leading to iron overload and lipid peroxidation [104].

Ferroptosis not only affects tumor cells but alters the tumor microenvironment, modulating immune cell function, and thus improving the efficacy of immunotherapy. One study identified N6F11 as a selective ferroptosis inducer that degrades GPX4 specifically in cancer cells without affecting immune cells, thereby triggering ferroptosis in cancer cells and initiating HMGB1-dependent antitumor immunity mediated by CD8^+^ T cells. The researchers further showed that N6F11 also enhances the efficacy of the immune checkpoint blockade in advanced cancer models, providing a promising strategy to boost ferroptosis-driven antitumor immunity [105].

Aspartate aminotransaminase (GOT1) plays a key role in the interconversion of glutamate and aspartate, regulating the intracellular amino acid balance and REDOX metabolism. One study found that inhibition of GOT1 disrupts amino acid metabolism and antioxidant defense systems, leading to oxidative stress and ferroptosis. The research also explored the potential of GOT1 inhibition as a novel therapeutic strategy for pancreatic cancer. Combining GOT1 inhibitors with other anticancer agents may enhance its therapeutic efficacy, particularly in cases where pancreatic cancer exhibits resistance to existing treatments, making GOT1 inhibition a potentially effective complementary strategy [106].

Microsomal glutathione S-transferase 1 (MGST1) is a membrane-bound transferase that prevents ferroptosis by binding to 5-lipoxygenase (5-LOX). Researchers found that MGST1 exerts an anti-ferroptotic effect in pancreatic cancer cells by inhibiting lipid peroxidation. Gene knockout experiments have shown that depletion of MGST1 promotes ferroptosis in pancreatic cancer cells. By contrast, the re-expression of MGST1 in cells restores their resistance to ferroptosis [107]. Therefore, MGST1 emerges as a promising therapeutic target, and targeting the REDOX-sensitive pathway of MGST1 may provide a novel strategy for treating pancreatic cancer. By inhibiting the effect of MGST1, it can enhance the sensitivity of pancreatic cancer cells to ferroptosis, thereby improving therapeutic efficacy.

Another study analyzed the interaction between CAFs and pancreatic cancer cells using conditioned media and co-culture systems. The study found that CAFs secrete cysteine to support GSH synthesis, helping tumor cells maintain antioxidant defenses. This mechanism is regulated through the TGF-β/SMAD3/ATF4 signaling pathway [108]. These findings suggest that targeting CAFs could serve as a novel strategy to overcome resistance to ferroptosis in pancreatic cancer.

Table 1 summarizes the newly discovered drugs/compounds or targets for regulating ferroptosis in pancreatic cancer. Overall, these studies focus on iron metabolism, lipid metabolism, and antioxidant defense systems as novel therapeutic approaches for pancreatic cancer, providing new potential targets and offering fresh insights into combating pancreatic cancer through ferroptosis. However, the clinical application of ferroptosis still faces significant challenges, particularly in selecting the appropriate drugs and enhancing their targeting capabilities to improve therapeutic efficacy and avoid systemic toxicity.

**Table 1:** Promising drugs/compounds or targets for regulating ferroptosis in pancreatic cancer.

Regulation	Drugs/Compounds or Targets	Mechanism	Effect	Cell Model	In Vitro/In Vivo Settings	Ref
Iron metabolism	Fento-1	Sequester lysosomal iron and induce membrane lipid oxidation	Ferroptosis inducer	CD44 high pancreatic cancer	In vivo	[93]
	Chimeras of salinomycin derivatives and the iron-reactive DHA	Sequester lysosomal iron and induce membrane lipid oxidation	Ferroptosis inducer	Drug-tolerant pancreatic cancer	In vitro	[96]
	*Atg5*/*Atg7* gene	Facilitate the formation of the autophagosome	Ferroptosis inducer	Panc1 and Panc 2.03	In vitro	[72]
	Zalcitabine	Induce mtDNA stress and ferritinophagy	Ferroptosis inducer	Panc1 and Capan2 cells	In vivo	[97]
	WJ460	Bind to myoferlin and trigger mitophagy	Ferroptosis inducer	BxPC3, Panc1, and MiaPaca-2 cells	In vivo	[98]
	DBDS	Impair mitochondrial structure and function, and liberate organelle-buffered iron	Ferroptosis inducer	Panc1, MiaPaca-2, AsPC1, and KP4 cells	In vivo	[100]
Lipid peroxidation	TMEM164	Mediate autophagosome formation	Ferroptosis inducer	Panc1 and KPC cells	In vivo	[101]
	miR-3173-5p	Inhibit ACSL4 expression	Ferroptosis inhibitor	Panc1 and BxPC3 cells	In vivo	[102]
	Fatostatin	SREBF1 inhibitor	Ferroptosis inducer	Primary PCa cell lines, Panc1, BxPC3, and MiaPaca-2 cells	In vivo	[109]
Antioxidant systems	ARID3A	Promote the transcriptional activity of PTEN and increase expression of GPX4	Ferroptosis inhibitor	MiaPaCa-2, Capan-1, Panc1, BxPC3, SW1990, AsPC-1, and HPDE cells	In vivo	[103]
	Thiostrepton	Inhibit GPX4 expression	Ferroptosis inducer	Panc1, MiaPaca-2, and BxPC3 cells	In vivo	[104]
	N6F11	Degrade GPX4 specifically	Ferroptosis inducer	Panc1 cell	In vivo	[105]
	GOT1 inhibitor	Disrupt amino acid metabolism	Ferroptosis inducer	Pa-Tu-8902, MiaPaca-2, and Capan-1 cells	In vivo	[106]
	MGST1	Bind to 5-LOX	Ferroptosis inhibitor	CFPAC1, Panc2.03, Panc1, and MiaPaca-2 cells	In vivo	[107]
	Simvastatin	Reduce the abundance of the MVP-derived CoQ	Ferroptosis inducer	Panc1, MiaPaca-2, AsPC1, and KP4 cells	In vivo	[100]

Abbreviations: DHA: dihydroartemisinin; *Atg5*/*Atg7*: autophagy-related genes; mtDNA: mitochondrial DNA; WJ460: a compound; DBDS: diphenyl diselenide; TMEM164: transmembrane protein 164; miR-3173-5p: a miRNA; ACSL4: acyl-CoA synthetase long-chain family member 4; SREBF1: sterol regulatory element-binding transcription factor 1; ARID3A: AT-rich interactive domain-containing protein 3A; PTEN: phosphatase and tensin homolog; GPX4: glutathione peroxidase 4; N6F11: a compound; GOT1: aspartate aminotransaminase; MGST1: Microsomal glutathione S-transferase 1; 5-LOX: 5-lipoxygenase; MVP: mevalonate pathway; CoQ: coenzyme Q.

## 4. Ferroptosis Therapy Based on Nanotechnology: Beyond Drug Delivery

**Figure 3:**
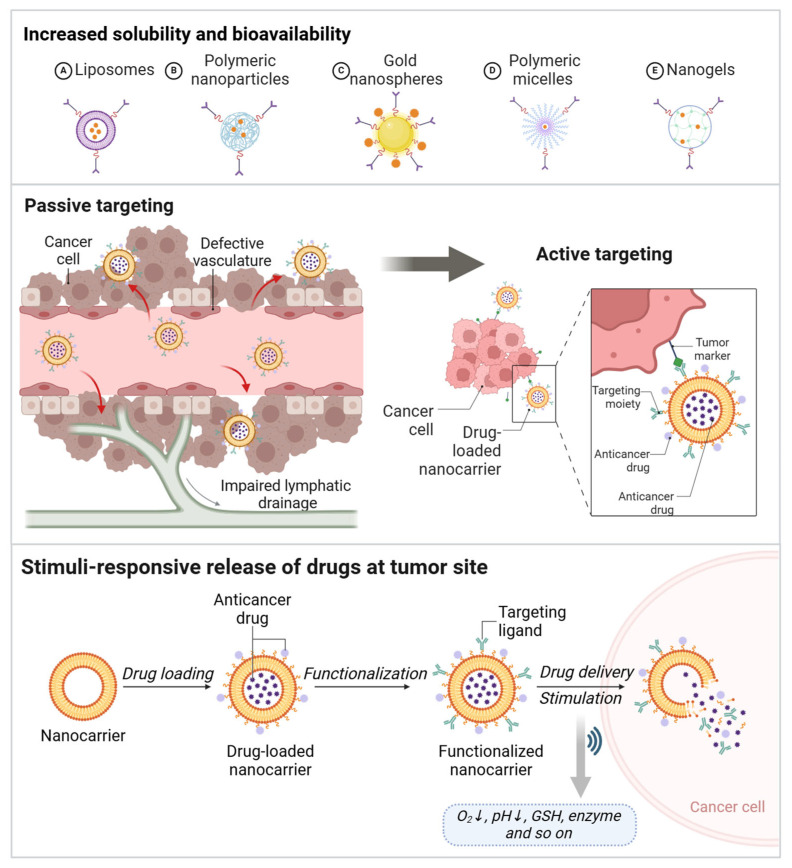
The advantages of nanotechnology for cancer treatment (the downward-pointing arrows indicate a decrease). Created in BioRender. Xiaoyan, Y. (2025) https://BioRender.com/hwol98x. Abbreviations: O_2_: oxygen; pH: power of hydrogen; GSH: glutathione.

Nanotechnology encompasses the design, manipulation, and application of materials at the nanoscale, typically ranging from 1 to 100 nm. By leveraging the unique physicochemical properties of nanomaterials, nanotechnology enables the creation of novel functionalities and characteristics, such as surface effects and quantum effects that differ from those of macroscopic materials [110]. Owing to this and taking advantage of the advancements in nanotechnology, nanomedicine offers many benefits that are unattainable with conventional pharmaceuticals (Figure 3) [111,112]. First, nanoparticles effectively encapsulate and deliver anticancer drugs, improving their solubility, stability, and bioavailability. In pancreatic cancer treatment, nanoparticle-based drug delivery systems can overcome the common drug resistance issues associated with conventional chemotherapy by enhancing drug penetration and delivery on one hand, and inhibiting the metabolic degradation of chemotherapeutic agents on the other hand [113,114,115]. Second, by designing nanoparticles with specific targeting capabilities, anticancer drugs can be precisely delivered to tumor sites, improving drug bioavailability and therapeutic efficacy while reducing side effects on normal tissues. For instance, surface-modified nanoparticles can bind to specific receptors on cancer cell membranes, enhancing the specificity of drug delivery [116,117]. Third, the utilization of nanomedicine for stimulus-responsive drug release represents a significant advancement in contemporary nanomedicine delivery systems, particularly in the realms of precision drug delivery and targeted therapy. Leveraging the controllable properties of nanocarriers enables drugs to be released specifically in response to the unique environmental conditions at the lesion site (such as temperature, pH, light, magnetic fields, enzymes, etc.). This approach enhances therapeutic efficacy while minimizing adverse effects on healthy tissues [118,119]. In addition to functioning as an individual drug delivery system, nanomedicine can also execute multiple functionalities. For instance, nanomedicine is capable of simultaneously delivering therapeutic agents, such as drugs, genes, and antibodies, or integrating treatment with diagnosis (e.g., theranostic nanomedicine) to implement multifaceted therapeutic strategies.

### 4.1. Nanotechnology-Induced Ferroptosis in Pancreatic Cancer

Despite the exploration of several small-molecule inducers for triggering ferroptosis, their clinical applications are hindered by short half-lives in circulation, rapid renal clearance, poor selectivity, and limited endogenous iron availability. For instance, erastin demonstrates unstable metabolic properties and inadequate water solubility, leading to more pronounced side effects [7]. Compounds such as sulfasalazine, sorafenib, cisplatin, and artemisinin are currently regarded as promising ferroptosis inducers; however, their clinical utility is constrained by severe adverse drug reactions, non-specific biodistribution, and the necessity for high dosages [120,121]. Therefore, therapeutic strategies should be engineered to selectively target cancer cells while minimizing the impact on healthy cells. Nanotechnology-induced ferroptosis has emerged as a promising alternative to enhance the efficacy of pancreatic cancer therapies. This section will highlight the latest advancements in nanotechnology-based ferroptosis therapy for the treatment of pancreatic cancer (Table 2).

Iron oxide nanoparticles are the most widely used exogenous iron-delivery nanomaterials, capable of directly releasing iron ions into cells. Since bare iron oxide nanoparticles tend to aggregate via dipole–dipole interactions, a surface coating is necessary to enhance their colloidal stability and dispersibility. This improves blood circulation time and cellular uptake while minimizing nonspecific interaction. Dextran is one of the most commonly used coatings, as it can be covalently crosslinked with amine-containing compounds, facilitating the conjugation of drugs and ligands. In one study, the researchers exposed PANC-1 cells to dextran-coated γ-Fe_2_O_3_ nanoparticles (DIO-NPs) at different concentrations (14, 28, 42, 56 μg/mL) for up to 72 h. The results showed that DIO-NPs exhibited biocompatibility at a low dose of 28 μg/mL, whereas exposure to a high dose of 56 μg/mL reduced PANC-1 cell viability to 50% after 72 h [122]. This occurs through cellular internalization, where the NPs decompose in the acidic microenvironment of lysosomes and release Fe^2+^/Fe^3+^ ions into the cytoplasm. These ions can then react with H_2_O_2_ to generate highly reactive ·OH, ultimately leading to ferroptosis.

**Figure 4:**
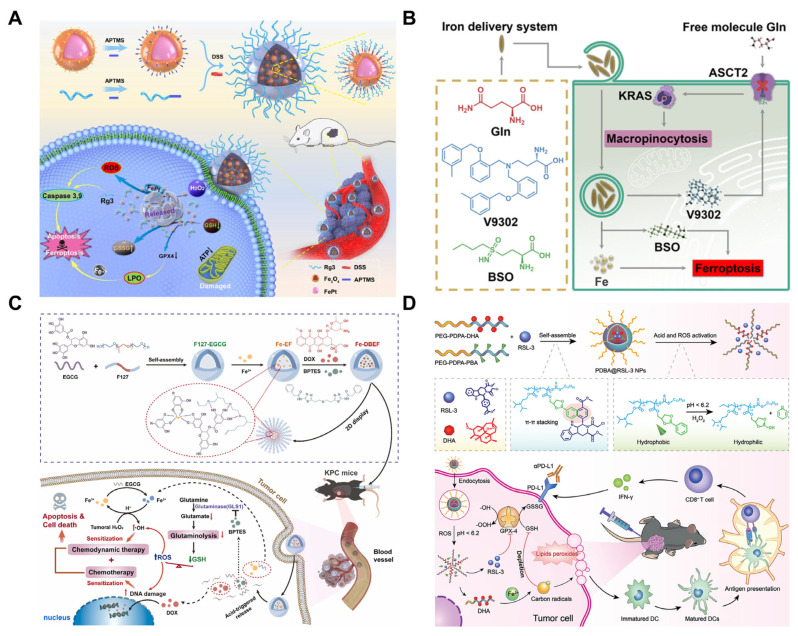
Nanotechnology-induced ferroptosis in pancreatic cancer. (**A**) Preparation of Nano-FePt-Rg3 (NFPR) and treatment mechanism. Copyright 2022, with permission from Elsevier [123]. (**B**) Preparation of glutamine-modified iron delivery system and treatment mechanism. Copyright 2024, with permission from the American Chemical Society [125]. (**C**) Preparation and treatment mechanism of Fe-DBEF NPs. Copyright 2024, with permission from Elsevier [126]. (**D**) Preparation and treatment mechanism of PDBA@RSL-3. Copyright 2022, with permission from John Wiley and Sons [127].

In the Fenton reaction, Fe^2+^ exhibits a reaction efficiency that is several dozen times higher than that of Fe^3+^. However, Fe^2+^ is highly susceptible to various endogenous factors before reaching the tumor site. Therefore, effectively protecting Fe^2+^ from oxidative stress before it arrives at the tumor, and ensuring its controlled release at the right time and location in vivo is of critical importance. Achieving this goal will also help to mitigate the toxicity caused by excessive Fenton-like heavy metals in ferroptosis. To overcome this challenge, a multilayered nano-drug (Nano-FePt-Rg3, NFPR) with a hierarchical microstructure was designed and synthesized by coupling (FePt)@(Fe_1−x_Ptx)Oy(OH)z hybrid NPs with ginsenoside Rg3 [123]. Due to the acid sensitivity of NFPR, the surface modification with Rg3 significantly altered the pharmacokinetics of systemically administered NPs, enhancing tumor accumulation by prolonging blood circulation time and enabling dynamic disassembly within the tumor microenvironment. This strategy effectively prevents the premature leakage of metal ions before reaching the tumor site, ensuring their precise release at the correct time and location in vivo. The Fe^2+^ and Fe^3+^ ions on the surface of the nanoparticles, under the catalytic action of FePt, react with H_2_O_2_ to generate a large amount of ROS, thereby activating ferroptosis (Figure 4A). This study provides new insights into the exploration of dynamic nano-catalysts for safe and effective anticancer therapy.

Due to the heterogeneous reconfiguration of glutamine addiction metabolism in pancreatic cancer [124], glutamine modification can be considered a source of effective endocytosis of glutamine nutrients in pancreatic tumor cells, thereby enhancing endocytosis of iron-loaded carriers and promoting efficient intracellular iron delivery. Based on this, the researchers introduced a novel glutamine-modified iron delivery system (IDS), which was synthesized by hydrolyzing ferric chloride in an aqueous solution containing amino acids and amino acid-like reagents, resulting in the formation of Fe^3+^/amino acid complex-coated β-FeOOH nanoshuttles (FeOOH@Fe/AA NSs). The adjustable composition of the amino acid components enabled the incorporation of glutamine, V9302 (a glutamine transporter inhibitor), and l-buthionine sulfoximine (BSO, a ferroptosis inducer). Meanwhile, efficient exogenous iron delivery combined with supplemental BSO significantly improved the therapeutic efficacy of IDS against pancreatic cancer through ferroptosis induction (Figure 4B) [125].

Research has demonstrated that the inhibition of glutaminase (GLS1) can effectively block the pathway of glutamine metabolism and induce GSH depletion. Consequently, researchers have developed an efficient assembly strategy to synthesize a novel metal–polyphenol-based multifunctional nanodrug (Fe-DBEF). This nanodrug consists of Pluronic F127-stabilized iron-ion-crosslinked epigallocatechin gallate (EGCG) nanoparticles loaded with a GLS1 inhibitor and the chemotherapeutic drug doxorubicin (DOX). Fe-DBEF induces synergistic ferroptosis by delivering exogenous iron and depleting GSH in tumor cells. In vitro cell experiments, human organoid models, and KPC mouse models demonstrated that Fe-DBEF exhibits potent antiproliferative effects against pancreatic cancer (Figure 4C) [126].

Pancreatic cancer exhibits resistance to radiotherapy and chemotherapy, a process in which cancer stem cells (CSCs) and activated pancreatic stellate cells (PSCs) play a pivotal role by driving tumor stemness properties [128]. Diethyldithiocarbamate (DE), the active metabolite of the FDA-approved anti-alcoholism drug disulfiram, irreversibly inhibits aldehyde dehydrogenase 1A1 (ALDH1A1). This enzyme is pivotal for maintaining CSCs’ stemness, as it protects CSCs from oxidative stress, suppresses apoptosis, and enhances chemoresistance. Beyond DE-mediated apoptosis, ferroptosis has emerged as a promising target for eradicating stemness properties and overcoming drug resistance by disrupting cellular redox homeostasis. Consequently, the researchers developed a nanoformulation of DE using ferrous oxide nanoparticles (FeO NPs-DE) [129]. The selective accumulation of FeO NPs and DE induces lethal lipid peroxidation accumulation, ultimately leading to the collapse of CSCs and PSCs. Both the established 3D co-spheroid in vitro and in vivo models demonstrated that FeO NPs-DE exhibits superior antitumor efficacy compared to gemcitabine. This potent effect is attributed to the selective accumulation of FeO NPs-DE in tumor tissues, which induces iron-dependent ferroptosis.

The classical Fenton reaction involves the Fe^2+^-catalyzed decomposition of H_2_O_2_ to generate highly reactive ·OH. The Fenton-like reaction, on the other hand, refers to a similar oxidation process that typically employs non-iron metal ions, such as Cu^+^ or Mn^2+^, to facilitate the production of ·OH from H_2_O_2_ [130,131]. It has been reported that the Fenton-like reaction exhibits higher catalytic efficiency compared to iron-catalyzed Fenton reactions [132]. Therefore, an increasing number of studies have focused on utilizing Cu^+^ or Mn^2+^ as catalysts to induce Fenton-like reactions to generate ·OH. Dihydroartemisinin (DHA) is a sesquiterpene lactone characterized by its intramolecular peroxide bridge structure, and is widely utilized in clinical anti-malarial therapy. The research into the mechanism of DHA’s antimalarial activity suggests that its lethal effect on Plasmodium may be attributed to the reaction between the internal peroxide bridge of DHA and Fe^2+^ from heme. Consequently, the researchers combined DHA with MnO_2_ to activate the Fenton-like reaction catalyzed by Mn^2+^ to produce ·OH and induce ferroptosis. They constructed a nanoparticle platform (MLP@DHA&Ce6) by co-loading DHA and the photosensitizer Ce6 in lipid-supported MnO_2_ nanoparticles. MLP@DHA&Ce6 exhibited well retention in tumors within 48 h and MnO_2_ nanozymes gradually decomposed in the tumor microenvironment, releasing Mn^2+^ ions and O_2_. Their experimental results showed that the constructed nanoplatform can effectively induce ROS production, and tumor growth was almost completely suppressed in a mouse model of pancreatic cancer [133].

In addition to nanoparticles that directly deliver iron or other metal ions, nanoparticles can be used to deliver antioxidants, inhibiting the antioxidant defense system of tumor cells and thus promoting ferroptosis. Researchers have revealed that platelet-derived vesicles can efficiently encapsulate RSL-3 and deliver it to pancreatic cancer tissues via the inherent targeting properties of platelets. By inhibiting GPX4, RSL-3 prevents the clearance of LPOs and leads to ferroptosis. Moreover, RSL-3 exhibits anti-angiogenic effects, which can reduce tumor blood supply and inhibit tumor growth and metastasis. Both in vitro and in vivo studies have demonstrated significant therapeutic efficacy of platelet-vesicle-encapsulated RSL-3 (RSL-3@PVs) in treating pancreatic cancer. The tumor weight in the PBS group was twice that of the RSL-3@PVs group, where tumor growth was relatively slower, with the baseline tumor volume maintained at approximately 100–200 mm^3^. [134]. Another study developed a polymer micelle (PDBA@RSL-3 NPs) that responds to intracellular acidic environments and oxidative stress to co-deliver DHA and RSL-3. DHA can induce lipid peroxidation in tumor cells, while RSL-3 further exacerbates this effect by inhibiting GPX4. A flow cytometry analysis revealed that PDBA@RSL-3 NPs induced a 6.3-fold increase in lipid ROS accumulation in tumor cells compared to the PBS group. Neither PDB@RSL-3 (lacking DHA) nor PDBA nanoparticles (lacking RSL-3) showed significant inhibition of tumor growth, only exerting mild effects. By contrast, PDBA@RSL-3 NPs achieved approximately 50% suppression of tumor growth. The results demonstrated that the synergistic effect achieved by co-delivery of DHA and RSL-3 more effectively induced ferroptosis in pancreatic cancer (Figure 4D) [127]. MnFe_2_O_4_ is a nanomaterial with an enzyme-like activity that mimics the function of natural enzymes, such as peroxidase and glutathione peroxidase. This characteristic enables it to facilitate the generation of ROS and the depletion of GSH, ultimately inducing ferroptosis. Researchers have developed a novel nanosystem by incorporating gemcitabine into MnFe_2_O_4_-loaded carbonaceous (MFC) nanoparticles, resulting in MFC-Gem. Gemcitabine functions as a chemotherapy agent by inhibiting DNA synthesis, while MnFe_2_O_4_ induces ferroptosis through its enzyme-like activity. The synergistic effects of these two components overcome the drug resistance associated with gemcitabine monotherapy and enhance its cytotoxicity against cancer cells. Compared to the control group, monotherapy with either MFC or Gem exhibited suboptimal tumor suppression. By contrast, the MFC-Gem combination group showed minimal tumor growth without significant volume increase in a murine model of pancreatic cancer [135].

### 4.2. Nanotechnology-Mediated Synergistic Ferroptosis Strategies in Pancreatic Cancer

The advancement in the development of nanoparticles exhibiting distinctive functional characteristics, such as photothermal effects, photodynamic effects, imaging capabilities, and magnetothermal effects, has presented a promising opportunity to construct a multifunctional nanoplatform for cancer therapy [136,137,138,139,140]. To further enhance the efficacy of ferroptosis in pancreatic cancer treatment, researchers have initiated explorations into strategies that integrate ferroptosis with other therapeutic modalities. By combining ferroptosis with complementary treatments (such as chemotherapy, photothermal therapy (PTT), photodynamic therapy (PDT), cuproptosis, anti-stromal therapy, anti-angiogenesis therapy, etc.), synergistic effects can be achieved to augment therapeutic outcomes and address the limitations inherent in single-modality approaches.

**Figure 5:**
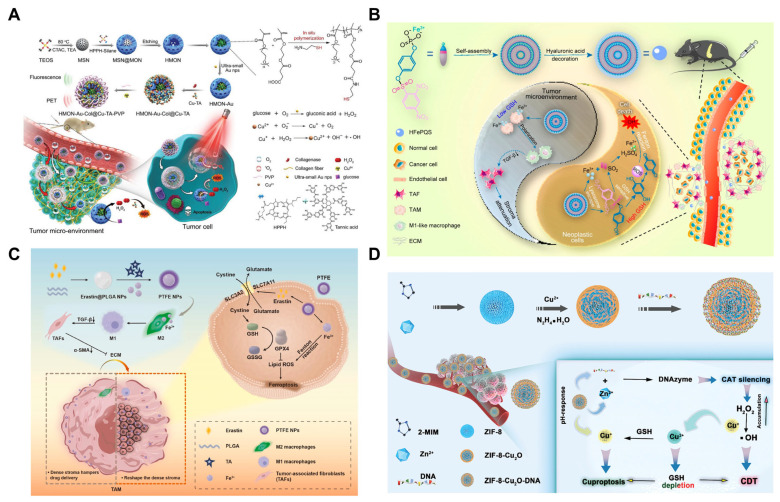
Nanotechnology-mediated synergistic combination strategies in pancreatic cancer. (**A**) Nanotechnology-mediated ferroptosis-PDT combination strategy. Copyright 2019, with permission from John Wiley and Sons [144]. (**B**,**C**) Nanotechnology-mediated stroma-targeted ferroptosis. (**B**) Copyright 2020, with permission from the American Chemical Society [147] (**C**) Copyright 2024, with permission from Elsevier [148]. (**D**) Nanotechnology-mediated ferroptosis-cuproptosis. Copyright 2023, with permission from John Wiley and Sons [149].

In recent years, PDT has garnered increasing attention as an innovative treatment modality in oncology. Upconversion nanoparticles (UCNPs), owing to their unique upconversion luminescence properties, can generate visible light upon near-infrared (NIR) excitation and serve as ideal carriers for PDT [141,142]. Consequently, researchers have proposed a novel strategy that integrates cancer cell membrane-coated UCNPs with Zn_x_Mn_1−x_S core–shell nanoparticles (BUC@ZMS) to achieve targeted PDT and ferroptosis combination therapy for pancreatic cancer. The UCNPs core can be excited by NIR light to produce high-energy visible or ultraviolet light, which subsequently activates the shell to generate ROS and inhibit tumor growth. The Zn_x_Mn_1−x_S shells can induce ferroptosis by generating ·OH via the Fenton-like reaction within tumor cells. As a natural biomembrane, the cancer cell membrane possesses excellent biocompatibility, immune evasion properties, and the ability to target tumor cells. After intravenous injection of Cy5.5-labeled nanocomposites (BUC@ZMS-Cy5.5), fluorescence imaging was performed to track the in vivo distribution of BUC@ZMS in mice. The BUC@ZMS group exhibited the strongest fluorescent signals at the tumor site, demonstrating its active tumor-targeting capability through homologous binding. Compared to other groups, the BUC@ZMS + 0.5 W cm^−2^ NIR group significantly inhibited tumor progression. Notably, as the NIR irradiation intensity increased, the BUC@ZMS + 1 W cm^−2^ NIR group demonstrated the greatest reduction in tumor growth [143]. In another study, an in situ polymerized hollow mesoporous silica nanoreactor (HMON) was designed as a biocatalyst carrier with enhanced ROS generation capability (Figure 5A). The HMON structure was formed via in situ polymerization within the nanoreactor, and the photosensitizer HPPH was incorporated into the framework to produce ROS and to facilitate PDT upon light activation. Additionally, ultra-small gold nanoparticles were loaded into the cavity to catalyze the conversion of glucose to H_2_O_2_, thereby supplying H_2_O_2_ for the Fenton reaction. Finally, Cu^2+^ was deposited on the HMON surface, which catalyzes the conversion of self-supplied H_2_O_2_ to ·OH through a Fenton-like reaction. In both in vitro and in vivo experiments, the nanoreactor effectively induced oxidative stress in tumor cells. The synergistic effects of PDT and ferroptosis led to the rapid regression of tumors in approximately 80% of mice, resulting in long-term tumor-free survival [144].

As previously introduced, a prominent pathophysiological hallmark of pancreatic cancer is the extensive deposition of the ECM, in which CAFs and TAMs play pivotal roles in both the ECM deposition and remodeling. Besides inducing ferroptosis via the Fenton reaction to eliminate tumor cells, iron ions can also induce TAMs to polarize into an M1 phenotype, thereby inhibiting CAF activation [145,146]. Consequently, to overcome the barrier posed by the tumor stroma while effectively killing tumor cells, researchers have developed a customized nanocomplex that simultaneously targets tumor cells and reprograms TAMs (Figure 5B). This nanocomplex can control the release of its components based on the varying REDOX environments within TAMs and tumor cells. In TAMs with low GSH levels, the nanocomplex selectively releases iron ions to induce TAM polarization. However, in tumor cells with a high GSH content, the nanocomplex triggers a robust Fenton reaction by releasing iron ions, hydroquinone, and SO_2_ in response to GSH, leading to effective tumor cell death. Thus, this study presents a novel strategy that integrates ferroptosis induction with anti-stromal therapy, achieving controlled cancer cell damage and reprogramming of the tumor microenvironment through customized nanocomplexes, thereby enhancing therapeutic outcomes for pancreatic cancer [147]. Another study developed a nanoparticle, designated as PTFE, which features a core–shell structure (Figure 5C). The core is composed of polylactic-glycolic acid (PLGA) copolymer encapsulating erastin, while the shell is formed by Fe^3+^ and tannic acid, creating a metal–organic framework (MOF). This design enables PTFE to effectively induce tumor cell death through the synergistic effect of ·OH generated from Fe^3+^ in Fenton reactions and erastin-induced lipid peroxidation. Additionally, the PTFE nanoparticles facilitate the repolarization of TAMs into M1-type macrophages, modulate the tumor microenvironment density, and enhance the accumulation and deep penetration of nanoparticles within tumors. The tumor penetration effect of PTEE was demonstrated in both in vitro multicellular tumor spheroid models and in vivo murine models of pancreatic cancer characterized by mesenchymal-rich subcutaneous tumors. Given PTFE’s dual advantages—efficient ferroptosis activation and deep tumor cell infiltration—the in vivo tumor inhibition rate reached 75% in tumor-bearing mouse models, highlighting its potential as a potent antitumor agent [148].

Carbon monoxide (CO) is regarded as a promising anticancer agent; however, its low concentration in the body and rapid clearance pose significant challenges for direct cancer treatment. To address this issue, researchers have developed a nanoplatform (CORM@GOx) capable of triggering CO release. CORM@GOx incorporates manganese carbonyl (MnCO) into zirconium-based metal–organic frameworks (Zr-MOFs), enabling CO release upon stimulation by H_2_O_2_. The glucose oxidase encapsulated within the nanoplatform catalyzes glucose oxidation, generating H_2_O_2_, which promotes CO release and depletes energy resources in tumor cells. Additionally, Mn^2+^ ions from MnCO react with H_2_O_2_ produced in tumor cells to generate cytotoxic ·OH, thereby enhancing the anticancer efficacy. In the in vivo anti-tumor efficacy study, CORM@GOx exhibited the most potent anti-tumor effects, strongly suppressing tumor progression with a remarkable tumor inhibition rate of 94.3%. Consequently, this nanoplatform exerts a triple anticancer effect through synergistic ferroptosis, CO gas therapy, and starvation therapy. Although this study employed a liver cancer model, its innovative combination therapy strategy also provides new insights for pancreatic cancer treatment [150].

Cuproptosis, recently described by Tsvetkov et al., represents a unique copper-dependent cell death pathway [151]. Distinct from apoptosis (caspase-dependent) and ferroptosis (iron-dependent lipid peroxidation), cuproptosis hinges on intracellular copper overload, which disrupts mitochondrial function via lipoylated protein aggregation and compromises energy metabolism by destabilizing iron–sulfur (Fe-S) cluster proteins in the tricarboxylic acid (TCA) cycle [152]. This groundbreaking mechanism has catalyzed a new wave of studies exploring copper as a therapeutic agent for targeted tumor eradication. One study designed a DNAzyme-based cascade nanoreactor (ZIF-8-Cu_2_O-DNA) to enhance ferroptosis and cuproptosis, which consists of a zeolitic imidazolate framework (ZIF-8) loaded with DNA, Zn^2+^, and Cu^+^ (Figure 5D). Upon entering the cell, ZIF-8-Cu_2_O-DNA releases DNA, Zn^2+^, and Cu^+^ in the mildly acidic environment of the tumor cells. The released DNA and Zn^2+^ form DNAzymes that specifically recognize and cleave catalase RNA associated with catalytic enzymes, resulting in the accumulation of H_2_O_2_. Simultaneously, the released Cu^+^ catalyzes the generation of ·OH from H_2_O_2_ via a Fenton-like reaction, thereby inducing ferroptosis. Additionally, the release of Cu^+^ effectively induces cuproptosis, accompanied by the generation of Cu^2+^. The resulting Cu^2+^ can be partially reduced back to Cu^+^ by GSH, forming a copper ion cycle that ensures a synergistic effect between ferroptosis and cuproptosis. Moreover, GSH depletion further enhances the efficacy of both ferroptosis and cuproptosis. The results demonstrated that, compared to ZIF-8 and ZIF-8-Cu_2_O, the cascaded nanoreactor ZIF-8-Cu_2_O-DNA exhibited the most pronounced tumor suppression effect. Compared to traditional therapies, this nanoreactor’s synergistic therapeutic strategy has shown superior therapeutic efficiency [149].

**Table 2:** Summary of studies on nanotechnology-based ferroptosis in pancreatic cancer.

Nanocarrier	Therapeutic Agents	Mechanisms	Effects	Cancer Model	In Vitro/In Vivo Settings	Ref
DIO-NPs	Fe^2+^	NPs decompose in the acidic microenvironment of lysosomes and release Fe^2+^/Fe^3+^ ions into the cytoplasm	Ferroptosis	PANC1 cells	In vitro	[122]
Nano-FePt-Rg3	Fe^2+^	Site-specific release of iron ions at the tumor and activates ferroptosis by Fenton reaction	Ferroptosis	L3.6pl cells	In vivo	[123]
FeO NPs-DE	Fe^2+^, DE	Induces iron-dependent ferroptosis potentiated by DE-mediated GSH and ALDH1A1 suppression	Ferroptosis	PANC1, MIA PaCa-2, and KPC cells	In vivo	[129]
Fe-DBEF	Fe^3+^, GLS1 inhibitor, and doxorubicin	Introduces iron and reduces GSH	Ferroptosis	Panc1 cells and KPC model	In vivo	[126]
FeOOH@Fe/Gln nanoshuttles	Fe^2+^	Enhances endocytosis of iron-loaded carriers and promotes efficient intracellular iron delivery	Ferroptosis	MiaPaCa2, SW1990, and PANC1 cells	In vivo	[125]
MnO_2_ nanoparticles	DHA, Mn^2+^, and Ce6	Induces the generation of ·OH by Fenton-like reaction	Ferroptosis	BxPC-3 Cells	In vivo	[133]
Platelet vesicles	RSL-3	Inhibits GPX4 activity	Ferroptosis	Panc1, Panc02, and Mia PaCa-2 cells	In vivo	[134]
Amphiphilic copolymer micelle	DHA and RSL-3	Induces lipid peroxidation and inhibits GPX4 activity	Ferroptosis	Panc02 cells	In vivo	[127]
Carbonaceous nanoparticles	Gemcitabine and MnFe_2_O_4_	Mimics the functions of natural enzymes to induce ferroptosis	Ferroptosis	Panc02 cells	In vivo	[135]
Chitosan nanoparticles	Gemcitabine	Decreases antioxidant capacity	Ferroptosis	CFPAC-1 cells	In vivo	[153]
Cu-MOF	Azo initiator AIPH	Induces the generation of ·OH by Fenton-like reaction and consumes GSH	Ferroptosis	Panc02 cells	In vivo	[154]
UCNPs and Zn_x_Mn_1−x_S core–shell nanoparticles	Mn^2+^	Induces the generation of ·OH by Fenton-like reaction and depletes intracellular GSH	Ferroptosis and PDT	Panc1 and BxPC-3 Cells	In vivo	[143]
HMON nanoparticle	HPPH photosensitizer, ultrasmall gold nanoparticles, and Cu^2+^	Provides self-supplied H_2_O_2_ and induces the generation of ·OH by Fenton-like reaction	Ferroptosis and PDT	BxPC-3 Cells	In vivo	[144]
Mn-doped Prussian blue nanoparticles	Mn^2+^	Induces the generation of ·OH by Fenton-like reaction	Ferroptosis and PTT	Panc1 cells	In vivo	[155]
ZIF-8	DNA, Zn^2+^, and Cu^+^	Upregulates intracellular H_2_O_2_ and induces the generation of ·OH by Fenton-like reaction	Ferroptosis and cuproptosis	Panc1 cells	In vivo	[149]
HFePQS nanocomplex	Fe^3+^, hydroquinone, and SO_2_	Induces the ferroptosis of cancer cells and the repolarization of TAMs	Ferroptosis and anti-stromal therapy	KPC cancer model	In vivo	[147]
PLGA nanoparticles	Erastin and MOF shell	Employs the Fe^3+^-induced Fenton reaction and inhibits System Xc-	Ferroptosis and anti-stromal therapy	KPC1199 cells	In vivo	[148]
Zr-based MOFs	MnCO and GOx	Provides self-supplied H_2_O_2_ and induces the generation of ·OH by Fenton-like reaction	Ferroptosis, starvation therapy, and CO gas therapy	HeLa cells	In vivo	[150]

Abbreviations: DE: diethyldithiocarbamate; ALDH1A1: aldehyde dehydrogenase 1A1; DHA: dihydroartemisinin; Ce6: chlorin e6; RSL-3: a compound; GPX4: glutathione peroxidase 4; GLS1: glutaminase; MOF: metal–organic framework; UCNPs: upconversion nanoparticles; GSH: glutathione; HPPH: 2-(1-hydroxyethyl)-chlorin(e)6; ZIF-8: zeolitic imidazolate framework; TAMs: tumor-associated macrophages; PLGA: polylactic-glycolic acid; MnCO: manganese carbonyl; GOx: glucose oxidase.

### 4.3. Endogenous Iron-Mediated Ferroptosis Therapeutics in Cancer Therapy

Compared with normal cells, tumor cells usually alter iron metabolism to increase intracellular iron accumulation in order to meet their metabolic needs, such as upregulating iron absorption and retention or reducing iron output [156,157]. Therefore, tumor cells rich in iron may be an innate resource that can trigger ferroptosis without exogenous iron input. Recent studies have shown that altering cellular iron metabolism (including iron absorption, storage, utilization and excretion) can lead to iron homeostasis disorder, thereby increasing the intracellular active iron content and causing ferroptosis [68,158,159]. Therefore, this new method of effectively triggering endogenous ferroptosis by mobilizing endogenous iron, both extracellular and intracellular, overcomes the limitations of relying on external iron sources, and points to a new direction for cancer treatment.

To date, there remain relatively limited investigations into the application of endogenous ferroptosis in pancreatic cancer therapy via nanotechnology. In a groundbreaking study, Kim et al. demonstrated for the first time that iron-free nanoparticles with iron-ion chelating capabilities (αMSH-PEG-C’ dots) possess the ability to induce ferroptosis in various pancreatic cancer cell lines [160]. The αMSH-PEG-C’ dots consist of Cy5 and ultrafine silica nanoparticles (C’ dots) as the core, coated with polyethylene glycol (PEG) and functionalized with a melanoma-targeting peptide (αMSH) on the outer layer. Mechanistic investigations reveal that the deprotonated surface of the C’ dots exhibits affinity for iron ions from the extracellular environment. Leveraging this unique property, the C’ dots can recruit and transport iron ions into tumor cells, thereby augmenting the labile iron pool within the cells. The subsequent accumulation of iron triggers ferroptosis in tumor cells, ultimately leading to inhibition of tumor growth. At the end of the study, the tumor volume in the treatment group was significantly reduced by 85% compared to the control group. This study demonstrates that utilizing nanomaterials as efficient promoters for recruiting extracellular endogenous iron represents a promising strategy to induce ferroptosis in pancreatic cancer.

Ferritin is regarded as the main iron storage protein in cells, and is overexpressed in various malignant tumors, including pancreatic cancer, hepatocellular carcinoma, and breast cancer. In recent years, a diverse range of nanotechnology-based drug delivery systems have demonstrated their efficacy in targeting and delivering ferritinophagy inducers to tumor cells. In the context of pancreatic cancer treatment, Yang et al. developed mesoporous silica nanoparticles (TreMMM), coated with manganese oxide and loaded with trehalose, which leveraged the ferritinophagy-dependent ferroptosis pathway for efficient tumor therapy [161]. Owing to the in situ growth of manganese oxide components on the nanoparticle surface, TreMMM depletes intracellular GSH. Simultaneously, the acidic pH environment and GSH depletion trigger the release of trehalose from TreMMM, thereby activating NCOA4-mediated autophagic degradation of ferritin to induce ferroptosis. Notably, this pioneering study on autophagy-induced ferroptosis utilizing nanotechnology offers promising insights into the treatment of pancreatic cancer.

**Figure 6:**
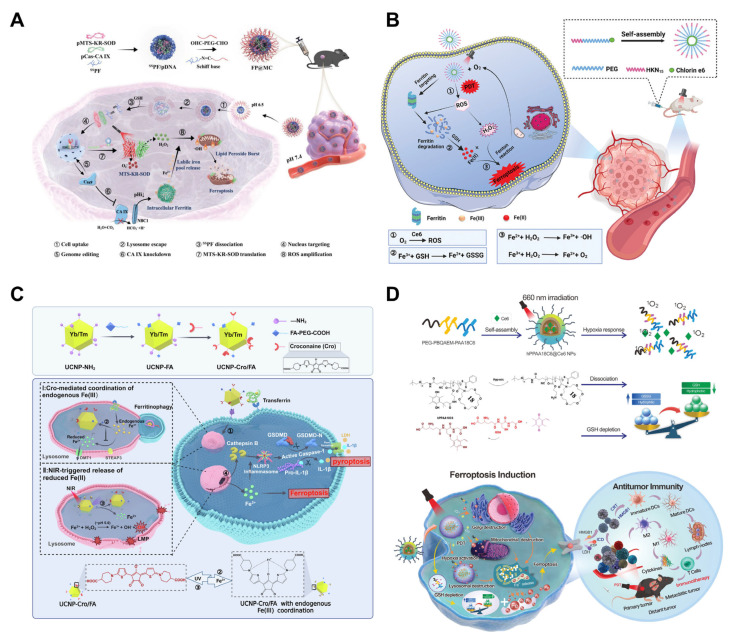
Endogenous iron-mediated ferroptosis therapeutics in cancer therapy. (**A**) The GSH/pH dual-responsive FP@MC nanosystem synergically treats tumors with PDT assistance for endogenous ferroptosis. Copyright 2023, with permission from John Wiley and Sons [162]. (**B**) Ferritin-hijacking nanoparticles Ce6-PEG-HKN_15_ for the synergistic PDT and endogenous ferroptosis therapy. Copyright 2022, with permission from John Wiley and Sons [163]. (**C**) Folic acid and croconaine functionalized upconversion nanoparticles (UCNP-Cro/FA) programmatically enhance endogenous iron-mediated lysosomal membrane permeabilization for tumor ferroptosis/pyroptosis therapy. Copyright 2025, with permission from Springer Nature [164]. (**D**) Polyvalent ferroptosis agonist hPPAA18C6@Ce6 hijacks endogenous iron and GSH to promote tumor immunotherapy. Copyright 2023, with permission from John Wiley and Sons [66].

Although nanotechnology-based endogenous iron-mediated ferroptosis holds great promise for cancer therapy, there remains a paucity of studies specifically addressing pancreatic cancer. Nevertheless, in recent years, numerous groundbreaking studies with significant scientific innovation have emerged in the context of other types of cancers. To provide novel insights and to stimulate innovative thinking for pancreatic cancer research, we have systematically summarized the representative studies published in recent years that focus on the application of nanotechnology-based endogenous iron-mediated ferroptosis in treating other types of cancers (Table 3). As previously discussed, ferritin serves as the primary iron storage protein in cells, and releasing the stored iron within it can promote the accumulation of labile iron pools, and thereby activate endogenous ferroptosis. In addition to the aforementioned method of releasing iron ions via ferritinophagy-induced autophagy, scientists have also investigated other novel approaches for liberating iron from ferritin. In a recent study, a GSH/pH dual-responsive nanocomplex (FP@MC) was developed (Figure 6A) [162]. This nanocomplex consists of fluorinated and cross-linked polyethyleneimine (PEI) with dialdehyde PEG layers, encapsulating MTS-KR-SOD and CRISPR/Cas9-CA IX. The knockdown of CA IX acidifies the intracellular microenvironment, thereby promoting ferritin-mediated iron release and initiating the endogenous ferroptosis pathway. Simultaneously, under 590 nm laser irradiation, MTS-KR-SOD generates substantial amounts of H_2_O_2_, exacerbating mitochondrial dysfunction and lipid peroxidation accumulation. The experimental findings demonstrate that, under light irradiation, FP@MC showed potent antitumor activity, reducing tumor volume to 204.4 ± 52.1 mm^3^, indicative of significant growth inhibition, offering a novel strategy for leveraging ferritin as an iron reservoir to enhance the treatment of ferroptosis-related cancers.

**Table 3:** Summary of recently reported endogenous ferroptosis inducers for cancer therapy applications. All experiments presented in the table were performed in vivo.

Endogenous Ferroptosis Inducers	Iron Source	Mechanism	Effects	Cancer Model	Ref
αMSH-PEG-C′ dots	Extracellular environment	Recruit iron from extracellular environment and promote its internalization	Ferroptosis	BxPC3 pancreatic cancer cells	[160]
TreMMM	Ferritin	Induce ferritinophagy and deplete intracellular GSH	Ferroptosis	PANC1 pancreatic cancer cells	[161]
FP@MC	Ferritin	Acidify the cytoplasm and de-hijack the labile iron pool from the ferritin	Ferroptosis	B16F10 melanoma cells	[162]
Ce6-PEG-HKN_15_	Ferritin	In situ destroy ferritin to release iron and deplete intracellular GSH	Ferroptosis and PDT	4T1 breast cancer cells	[163]
UCNP-Cro/FA	Lysosomes and ferritin	Sequester lysosomal iron and induce ferritinophagy, resulting in ROS-mediated LMP and leakage of lysosomal iron	Ferroptosis and pyroptosis	MCF-7 breast cancer cells	[164]
VC@^N3AM^cLAVs	Lysosomes	Enhance ROS production and boost LMP	Ferroptosis	CT26 colorectal cancer cells	[165]
DAR	Endo-lysosome	Rupture the lysosomal membrane by increasing mechanical strain and activating endogenous iron release	Ferroptosis and immunotherapy	4T1 and MCF-7 breast cancer cells	[166]
CuGA	Lysosomes	Hijack lysosomal iron and cause the release of Cu^+/2+^ and metal ion dysregulation	Ferroptosis, pyroptosis, and immunotherapy	4T1 breast cancer cells	[167]
PTO-Biotin Nps	Lysosomes	Induce lysosomal dysfunction-mediated Fenton reaction through photothermal effects	Ferroptosis	4T1 breast cancer cells	[168]
hPPAA18C6@Ce6	Mitochondria, lysosomes and Golgi	Release endogenous iron stored in the natural “iron pools” of cellular organelles and deplete GSH	Ferroptosis, PDT, and immunotherapy	B16F10 melanoma cells	[66]

Abbreviations: Fento-1: fentomycin-1; αMSH-PEG-C′ dots: alpha-melanocyte stimulating hormone (αMSH), polyethylene glycol (PEG); TreMMM: Tre-mSiO_2_@MnO_x_-mPEG; FP@MC: fluorinated and cross-linked polyethyleneimine (F), dialdehyde polyethylene glycol (P), mitochondria-targeting-sequence-KillerRed-Superoxide Dismutase (M), CRISPR/Cas9- Carbonic anhydrase IX (C); Ce6-PEG-HKN_15_: chlorin e6 (Ce6), polyethylene glycol (PEG), HKNKGKKNGKHNGWK polypeptide (HKN_15_); UCNP-Cro/FA: up-conversion nanoparticle (UCNP), croconaine molecules (Cro), folic acid (FA); VC@^N3AM^cLAVs: vitamin C (VC), N-(3-Aminopropyl) (N3AM), cross-linked lipoic acid vesicles (cLAVs); DAR: doxorubicin (D), tannic-acid (A), IR820 (R); CuGA: Cu-gallic acid; hPPAA18C6@Ce6: a hypoxia-responsive polymer bearing 18-crown-6 ring (hPPAA18C6), chlorin e6 (Ce6); PTO-Biotin Nps: phenothiazine-fused oxazine biotinylation nanoparticles; GSH: glutathione; ROS: reactive oxygen species; LMP: lysosomal membrane permeabilization.

In another study, the researchers conjugated the ferritin-targeting peptide HKN_15_ with the photosensitizer chlorin e6 (Ce6) to construct a ferritin-hijacking nanoparticle (CE6-PEG-HKN_15_), which could specifically target ferritin after cellular uptake (Figure 6B) [163]. Upon laser irradiation, the activated Ce6 within the nanoparticles releases iron by generating a substantial amount of reactive oxygen species (ROS) around ferritin, thereby disrupting the ferritin shell. The results demonstrated that these nanoparticles exhibited a potent tumor-killing effect through the synergistic combination of ferroptosis and PDT. In summary, this work underscores the potential of inducing endogenous ferroptosis via spatiotemporal disruption of ferritin, offering a model for ferritin iron-mediated ferroptosis anti-tumor therapy.

In subsequent studies conducted by this research group, the researchers not only leveraged the active iron storage within lysosomes but induced ferritinophagy, thereby further enhancing iron accumulation in lysosomes. In this study, the researchers synthesized an upconversion nanoparticle functionalized with folic acid and croconaine molecules (UCNP-Cro/FA) (Figure 6C) [164]. UCNP-Cro/FA mobilizes endogenous iron storage within cells through a two-step mechanism. First, Cro chelates with the abundant Fe^3+^ ions in lysosomes, inducing cytoplasmic iron depletion. In response to this depletion, cells initiate the autophagic degradation of ferritin, thereby promoting the accumulation of endogenous iron in lysosomes. Subsequently, under NIR irradiation, the ultraviolet (UV) light emitted by UCNP reduces the valence state of iron ions, leading to the rapid release of Fe^2+^ ions and triggering a robust Fenton reaction. Excitingly, this process induces lysosomal membrane rupture and the release of lysosomal contents, including iron ions and cathepsin B, which further triggers Caspase-1/GSDMD-mediated pyroptosis. Ultimately, the UCNP-Cro/FA + NIR group significantly inhibited tumor growth, extending the median survival time of mice by 16 days compared to the control group. This research offers novel insights into the comprehensive utilization of intracellular iron resources for therapeutic applications.

Table 3 also provides a summary of additional relevant studies focusing on the release of lysosomal iron resources to induce endogenous ferroptosis. These studies employ diverse methods to trigger the release of lysosomal iron, such as enhancing lysosomal membrane permeabilization (LMP) via reactive oxygen species (ROS) [165] or disrupting lysosomal membranes through mechanical stress [166]. In addition to ferroptosis treatments utilizing lysosomal iron, a recent study developed a multivalent ferroptosis agonist, hPPAA18C6@Ce6 (Figure 6D) [66]. This nanoagonist releases natural “iron pools” stored in various organelles, including lysosomes, the Golgi apparatus, and mitochondria. Furthermore, the positively charged primary amine of hPPAA18C6 can function as an immune adjuvant, promoting the maturation of dendritic cells and the activation of cytotoxic T lymphocytes. The findings demonstrated that hPPAA18C6@Ce6 achieved promising therapeutic outcomes in treating primary, distant, and metastatic tumors, offering an innovative strategy for the more complete utilization of intracellular natural iron reservoirs.

## 5. Pancreatic Cancer Imaging Based on Nanotechnology

Traditional imaging modalities encounter obvious limitations in the diagnosis of pancreatic cancer. Firstly, pancreatic cancer often presents with no discernible symptoms during its early stages, and the deep-seated location of a tumor within the pancreas poses challenges for detection. Conventional imaging techniques, such as CT, MRI, and ultrasound, struggle to identify small tumors at an early stage [169,170]. Moreover, these traditional methods typically provide only structural information, and fail to reveal the molecular characteristics and biomarkers of tumors, thereby limiting their utility in precise diagnosis and personalized treatment strategies. By contrast, imaging approaches based on nanotechnology exhibit distinct advantages. Certain nanoparticles inherently possess imaging capabilities due to their distinctive physical and chemical properties, such as gold nanoparticles, quantum dots, and iron oxide nanoparticles. By virtue of their intrinsic physical properties, these nanoparticles can directly engage in the imaging process, thereby diminishing the dependence on exogenous markers or supplementary contrast agents [171]. In addition, nanotechnology enhances imaging resolution through targeted delivery of nanoprobes to tumor cells, particularly facilitating the early detection of small lesions. Nanoprobes can be integrated with molecular imaging techniques to offer detailed molecular-level insights into tumors, including specific tumor markers and genetic mutations, aiding clinicians in comprehensively understanding tumor profiles and formulating personalized treatment plans [172,173,174]. Consequently, the research applications of various nanotechnology-based imaging modalities in pancreatic cancer will be introduced in this part (see Table 4).

### 5.1. Fluorescence Imaging

Fluorescence imaging generates visualizations by exciting fluorescent agents with specific light wavelengths to emit light. It offers advantages such as real-time imaging capability, high sensitivity, and low cost, making it particularly suitable for intraoperative superficial tumor margin delineation and vascular assessment. However, its limitations include shallow tissue penetration depth and susceptibility to interference, which may lead to false-positive results [175]. NIR dyes, such as indocyanine green [176], IR780 [177], and cyanine [178], have been extensively investigated for their applications in the fluorescence imaging of cancer. Quantum dots (QDs) are semiconductor nanomaterials that exhibit superior optical properties, including intense fluorescence, broad excitation spectra, and narrow emission peaks [179]. These characteristics enable QDs to effectively label specific molecules, cells, or tissues, rendering them invaluable in applications such as tumor imaging, cell tracking, and molecular imaging. For example, researchers have developed nanoparticles that conjugate graphene quantum dots (GQDs) with human serum albumin. These albumin-based nanoparticles exhibit no immunogenicity or hemolysis issues, possess excellent biocompatibility, and demonstrate negligible toxicity toward Panc-1 cells. Furthermore, in addition to the superior fluorescence properties of the synthesized GQDs, a commendable quantum yield of approximately 14% was achieved [180].

### 5.2. Magnetic Resonance Imaging (MRI)

Magnetic resonance imaging (MRI) utilizes a strong magnetic field to excite hydrogen nuclear resonance, acquiring signals to reconstruct high-resolution anatomical images. It offers advantages such as no ionizing radiation, superior soft-tissue resolution, and multi-parametric functional imaging capabilities. Clinically, MRI is widely applied, particularly for the detailed assessment of neurological structures, joints, and abdominal soft tissues. Although MRI eliminates ionizing radiation risks, gadolinium (Gd)-based contrast agents may induce nephrogenic systemic fibrosis (NSF) [181]. Therefore, their use should be exercised with caution in patients with renal insufficiency. Superparamagnetic iron oxide nanoparticles (SPIONs) are magnetic nanomaterials that have received extensive attention in recent years, which can not only improve MRI sensitivity but eliminate the risk of NSF associated with Gd-based contrast agents [182,183]. Furthermore, SPIONs can be utilized in magnetic hyperthermia treatments [182]. The conversion of energy absorbed from alternating magnetic fields into heat by SPIONs has been shown to be efficient. Studies have shown that this approach improves the survival of mouse models of pancreatic cancer [184]. Upconversion nanoparticles (UCNPs) represent a class of nanomaterials capable of emitting visible light under NIR irradiation. Their excellent biocompatibility and negligible autofluorescence render them highly suitable for bioimaging applications. Furthermore, UCNPs doped with the Gd element demonstrate superior relaxation properties, and can be used in MRI. Researchers have developed micelles based on UCNPs for targeted dual-modal imaging of pancreatic cancer. To achieve specific targeting of pancreatic cancer cells, the micelles were surface-modified with CD326-targeting antibodies, which can selectively bind to CD326 expressed on the surface of pancreatic cancer cells. Utilizing this targeted imaging micelle system, the researchers successfully achieved the accurate localization of tumor sites in a mouse model of pancreatic cancer [185]. In addition, manganese (Mn) is a paramagnetic metallic element that exhibits favorable relaxation properties. Consequently, Mn-based nanomaterials have garnered increasing attention for MR imaging in recent years [186,187,188].

### 5.3. Computed Tomography (CT)

Computed tomography (CT) imaging is an imaging technique used to diagnose diseases by detecting changes in the absorption of X-rays by different tissues. It offers advantages such as rapid acquisition speed, high skeletal resolution, and cost-effectiveness, making it the modality of choice for emergency trauma, pulmonary conditions, and vascular imaging. However, its limitations include exposure to ionizing radiation, poor soft-tissue contrast, and potential risks of iodinated contrast-induced allergies or nephrotoxicity. Metal nanoparticles exhibit obvious X-ray absorption properties, and can serve as effective CT contrast agents. Gold nanoparticles (AuNPs) exhibit great potential in cancer therapy and imaging owing to their distinctive physicochemical properties. The surface plasmon resonance effect of AuNPs enhances light scattering and absorption, positioning them as promising candidates for optical imaging applications [189]. Moreover, AuNPs possess significant X-ray absorption capabilities, which can markedly improve the image quality in CT scans by enhancing imaging contrast [190]. Researchers have developed a biomimetic nanoplatform based on AuNPs for targeted CT imaging for pancreatic cancer. The nanoplatform can actively target pancreatic cancer by coating the homologous cancer cell membrane on AuNPs. Based on the X-ray absorption effect of AuNPs, targeted CT imaging of pancreatic cancer was realized [191]. In addition to AuNPs, tantalum, tungsten, bismuth, and other metals have garnered increasing attention as potential contrast agents for CT [186]. For example, the researchers functionalized the surface of tantalum oxide nanoparticles with silane derivatives and conjugated fluorescence molecules and PEG, thereby endowing the nanoparticles with dual-mode CT and fluorescence imaging capabilities [192].

### 5.4. Ultrasonic Imaging

Ultrasonic imaging is an advanced diagnostic technique that employs high-frequency sound waves to detect reflected signals, thereby generating detailed images. It offers distinct advantages, including radiation-free operation, safety and portability, making it the preferred modality for obstetric applications, abdominal organ assessment, and superficial organ examinations. However, its limitations lie in significant signal attenuation by gas and bone tissues, suboptimal imaging of deep structures, and a strong dependence on operator expertise for diagnostic accuracy. Nanotechnology can enhance the resolution and penetration depth of ultrasonic images. Microbubbles, as effective ultrasound contrast agents, amplify the reflection of sound waves, thereby improving image contrast. They are widely utilized in the detection of blood flow dynamics, tumor characterization, and organ lesions. Researchers have engineered a hybrid nanomicelle system composed of lipid and polymer materials, conjugated with CD133 antibodies to specifically target pancreatic cancer cells. This nanomicelle is loaded with fluorescent dye FITC and perfluorohexane (PFH) with liquid–gas phase transition properties. Fluorescence imaging results demonstrated that the nanomicelles selectively bind to CD133-positive pancreatic cancer cells, with higher fluorescence signals at tumor sites compared to normal tissues. Ultrasonic imaging tests have revealed that the accumulation of nanomicelles in the tumor region markedly enhances ultrasonic echo signals, improving imaging resolution and facilitating precise tumor localization. Under the treatment of high intensity focused ultrasound (HIFU), the tumor volume of mice in the CD133-targeted nanomicelle treatment group showed a significant reduction. These findings indicate that CD133-targeted hybrid nanomicelles can effectively enhance the accuracy of pancreatic cancer imaging and improve the targeting efficacy of HIFU therapy. By integrating fluorescence/ultrasound imaging techniques with HIFU, the nanomicelles not only accurately locate tumors but minimize damage to surrounding healthy tissue [193].

### 5.5. Positron Emission Tomography (PET)

PET imaging technology leverages the distribution and metabolic characteristics of radioactive tracers to visualize disease states [194]. The utilization of PET in oncological imaging offers substantial advantages, particularly in the early detection and staging of tumors, as well as in monitoring therapeutic responses [195]. A key advantage lies in its capability to identify cancer at an earlier stage compared to conventional imaging modalities, such as CT and MRI, by assessing the metabolic activity of tumor cells. However, PET also presents certain limitations in cancer imaging. Firstly, ^18^F-fluorodeoxyglucose-based PET does not demonstrate a significant advantage in the early detection of pancreatic cancer [195,196]. Secondly, PET imaging can yield false positives due to various factors, including inflammation, infection, benign tumors, or non-neoplastic lesions. However, when integrated with nanomaterials, the specificity and resolution of PET imaging can be improved. Radioactive nanoparticles, including radiolabeled gold and silver nanoparticles, enable precise localization of radioactive substances to tumor or lesion sites, thereby improving the sensitivity and resolution of PET imaging. Additionally, nanocarriers, such as liposomes and polymer capsules, can be loaded with radioactive tracers, facilitating targeted imaging when combined with PET technology. PET/CT, as a highly sensitive molecular imaging modality, can precisely identify tumor-associated markers through the use of specific antibodies or ligands, thereby enhancing diagnostic accuracy. Researchers have conjugated AuNPs with targeted antibodies and labeled these AuNP-antibody complexes with ^89^Zr isotopes. In one study, pre-treatment with clodronate liposomes was employed to reduce the uptake of nanomaterials by the mononuclear phagocyte system, thereby improving the imaging efficacy for pancreatic tumors. The study evaluated the biodistribution of the probe in mice and its tumor-targeting capability. The results demonstrated a significant accumulation of the ^89^Zr-labeled AuNP-Ab complex in the pancreatic tumor region, indicating a pronounced tumor-targeting imaging effect. The combination strategy of clodronate liposomes with imaging probes enhances the tumor-targeting efficiency of AuNPs and improves the imaging performance for pancreatic tumors [197].

### 5.6. Multimodal Imaging

Although imaging techniques, such as CT, MRI, and ultrasound, are widely used in the diagnosis of pancreatic cancer, the sensitivity and specificity of traditional imaging methods are still limited due to the deep anatomical location of the pancreas and the heterogeneity of pancreatic tumors. Multimodal imaging combines the benefits of multiple imaging techniques, thereby offering comprehensive tumor information, including morphological, functional, and molecular characteristics. Based on this, the researchers developed a novel gold nanorod-based (AuNR) multimodal imaging contrast agent for MRI, CT, and photoacoustic imaging (PAI) of pancreatic cancer. The nanocontrast agent consisted of AuNR as the core, SiO_2_ as the hollow layer, and a lanthanide Gd carbonate shell. The experimental results showed that the nanocontrast agent has better relaxation attenuation than a Gadovist contrast agent in MRI imaging, and has greater X-ray attenuation than Visipaque in CT imaging. This study demonstrated the potential of nanomaterials to improve the sensitivity and specificity of multimodal imaging of pancreatic cancer [198].

**Table 4:** Summary of studies on nanotechnology-based imaging in pancreatic cancer. All experiments presented in the table were performed in vivo.

Nanocarrier	Targets	Imaging Agents	Effects	Cancer Model	Ref
Albumin nanoparticles	HA	GQDs	Fluorescence imaging	Panc-1 cells	[180]
Mesoporous silica nanoparticles	FA	Cy7.5	Fluorescence imaging	BxPC-3 cells	[178]
HAS-GEM/IR780 nanocomplexes	-	IR780	Fluorescence imaging	BxPC-3 cells	[199]
Dex-g-PCL/SPIO nanoparticles	Enolase 1	SPION	MRI	CFPAC-1 cells, Miapaca-2 cells	[200]
CD326-conjugated micelles	CD326	UCNPs	MRI	BxPC-3 cells	[185]
Collagenase-functionalized biomimetic Au NCs	Cancer cell membrane	AuNCs	CT	BxPC-3 cells	[191]
CD133-targeted nanomicelles	CD133	PFH	Ultrasonic imaging	BxPC-3 cells	[193]
Paclitaxel-loaded PFP nanoemulsions	-	PFP	Ultrasonic imaging	Miapaca-2 cells	[201]
Antibody-gold nanoparticle conjugate	5B1 antibody	^89^Zr	PET/CT	BxPC-3 cells	[197]
Gd-Au nanoclusters	Glypican-1	Gd, Au	Fluorescence imaging, and MRI	COLO-357 cells	[202]
Dextran-coated SPION	uMUC1	SPION, Cy5.5	Fluorescence imaging, and MRI	KCM pancreatic cancer model	[203]
Core–shell AuNR	-	AuNR, Gd	Photoacoustic imaging, MRI, and CT	KPF pancreatic cancer model	[198]

Abbreviations: HA: hyaluronic acid; GQDs: graphene quantum dots; FA: folic acid; SPION: superparamagnetic iron oxide nanoparticle; MRI: magnetic resonance imaging; UCNPs: upconversion nanoparticles; AuNCs: gold nanocages; CT: computed tomography; PFH: perfluorohexane; PFP: perfluoropentane; uMUC1: mucin 1; AuNR: gold nanorod.

## 6. Nanotechnology-Based Imaging-Guided Ferroptosis Therapy: Bridging Diagnosis and Treatment

Image-guided therapy combines different medical imaging techniques with treatment modalities to monitor tumor changes in real time and to provide more accurate treatment options [204,205]. Based on the advantages of nanotechnology, researchers can integrate therapeutic drugs and contrast agents with imaging properties into a nanoplatform, so that the treatment process can be monitored in real time. With the growing understanding of ferroptosis, combining this mechanism with nanotechnology and image-guided therapy into a multidimensional treatment strategy holds promise for providing more precise and effective therapeutic approaches for pancreatic cancer. Therefore, this part will summarize the cutting-edge advances of image-guided ferroptosis therapy based on nanotechnology in the treatment of pancreatic cancer.

### 6.1. Fluorescence Imaging-Guided Ferroptosis Therapy

NIR fluorescence optical imaging provides real-time tumor information, enabling surgeons to maximize tumor resection while minimizing damage to surrounding healthy tissue. With the continuous advancement of imaging technologies, sensitive fluorescence imaging techniques are now capable of monitoring the biochemical composition within tumors. Cysteine is an essential amino acid required for the synthesis of GSH. Therefore, real-time monitoring of cysteine levels is of significant importance for understanding the mechanisms of ferroptosis. Researchers have designed an NIR fluorescence probe based on a D-A-D (donor–acceptor–donor) structure, where the electronic interaction between the donor and the acceptor generates a fluorescence response under specific conditions. This probe is capable of emitting fluorescence at a wavelength of 717 nm in response to cysteine, enabling the real-time monitoring of cysteine fluctuations. They use this probe to track cysteine levels in pancreatic cancer cells during ferroptosis induced by erastin and RSL3, revealing differences in cysteine level changes between distinct ferroptosis pathways. Therefore, this study provides a novel monitoring platform for investigating the mechanisms of ferroptosis [206]. Compared to MRI and CT, fluorescence imaging requires relatively simple equipment and has higher imaging sensitivity. Consequently, the development of nanosystems for optically image-guided ferroptosis therapy in pancreatic cancer holds significant potential to enhance overall treatment outcomes.

### 6.2. MR Imaging-Guided Ferroptosis Therapy

**Figure 7:**
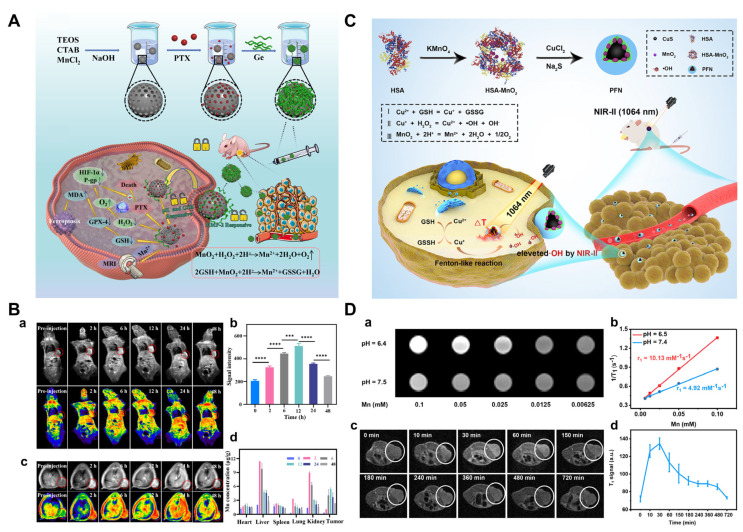
MR imaging-guided ferroptosis therapy based on nanotechnology. (**A**) Schematic representation of the preparation process and mechanism of action for Ge-Mn-MSN@PTX. (**B**) In vivo MRI and quantification of MRI signal intensity of mice after injection of Ge-Mn-MSN@PTX. MR imaging (**a**,**c**) (red circles indicate the tumor locations.) and quantitative MR signal intensity analysis (**b**) in nude mice within 48 h post Ge-Mn-MSN@PTX administration (**** *p* < 0.0001, *** *p* < 0.001); (**d**) In vivo biodistribution of Mn element within 48 h following Ge-Mn-MSN@PTX injection. Copyright 2024, with permission from Elsevier [207]. (**C**) Schematic of the preparation of PFN. (**D**) In vitro and in vivo MRI effect of PFN. (**a**) In vitro T_1_-weighted MRI images of PFN at different concentrations under pH 6.4 and pH 7.5 conditions; (**b**) Corresponding longitudinal relaxation rates (r_1_); (**c**) In vivo T_1_-weighted MRI images of PFN-administered groups at various time points (white circles indicate tumor locations); (**d**) Signal intensities of tumors in PFN-treated tumor-bearing mice at corresponding time points. Copyright 2020, with permission from Elsevier and the American Chemical Society [132].

Sensitive MRI is a powerful tool for the clinical diagnosis of pancreatic cancer, and the co-delivery of MR contrast agents with chemotherapeutic drugs and ferroptosis inducers under the guidance of MRI holds great potential for treating pancreatic cancer. In this context, researchers have proposed a novel nanodrug delivery system based on gelatin-coated, manganese-doped mesoporous silica nanoparticles (Ge-Mn-MSN) loaded with paclitaxel (PTX) (Figure 7A), a commonly used chemotherapeutic agent for pancreatic cancer. However, the therapeutic efficacy of paclitaxel is often limited by its poor penetration into tumor tissues and the development of drug resistance. By encapsulating paclitaxel in mesoporous silica nanoparticles, the drug’s concentration at the tumor site can be enhanced, and controlled drug release can be achieved. The surface of the nanoparticles is coated with gelatin via glutaraldehyde cross-linking, which imparts excellent biocompatibility, low hemolytic activity, and enzyme-responsive degradation, thus enabling the system to release the drug in response to the tumor microenvironment. Manganese (Mn) is incorporated into the system to perform multiple functions. First, Mn catalyzes the conversion of H_2_O_2_ to O_2_, effectively alleviating hypoxic conditions. Second, Mn can deplete intracellular GSH, thereby promoting lipid peroxidation and inducing ferroptosis. Additionally, Mn enables real-time MRI monitoring to evaluate treatment efficacy. With MRI monitoring, nanoparticle distribution and drug release can be dynamically observed, providing precise real-time feedback for therapy (Figure 7B). The experimental results demonstrated that the Ge-Mn-MSN@PTX nanoparticles exhibited favorable biosafety, anti-tumor activity, controllable drug release, and imaging tracking capabilities. The system’s controlled drug release, targeted delivery, and imaging monitoring offer a more accurate and efficient clinical treatment strategy, showing great potential in pancreatic cancer therapy [207].

In another study, the researchers engineered and synthesized a novel photothermal Fenton nanoreactor for synergistic photothermal therapy and ferroptosis guided by activatable MRI (Figure 7C). The nanoreactor (PFN) comprises three key components: manganese dioxide (MnO_2_), copper sulfide (CuS), and human serum albumin. Upon intravenous administration, the nanoreactor effectively accumulates at the tumor site due to its small size and enhanced permeability and retention (EPR) effect. Once within the tumor microenvironment, MnO_2_ degrades into Mn^2+^ ions under acidic conditions, enhancing T1-weighted MRI contrast and providing real-time imaging feedback for treatment monitoring (Figure 7D). CuS serves as a catalyst for Fenton-like reactions, which efficiently catalyzes the generation of ·OH, thereby inducing ferroptosis. In addition, CuS can absorb light energy at 1064 nm and convert it into thermal energy, generating localized hyperthermia. This photothermal effect can not only effectively kill tumor cells but improve the efficiency of Fenton-like reaction. Complete eradication of tumor growth was achieved in PFN-injected mice following laser irradiation, and no signs of recurrence were detected. In summary, this innovative photothermal Fenton nanoreactor integrates NIR-II photothermal therapy with ferroptosis, achieving precise therapeutic monitoring via MRI guidance. This synergistic treatment strategy offers a promising approach for pancreatic cancer therapy with substantial clinical potential [132]. It is crucial to highlight that MRI-guided treatment for pancreatic cancer is increasingly gaining prominence in clinical practice. Moreover, clinically approved MRI nanocontrast agents, such as iron oxide nanoparticles, are currently available. Consequently, the development of MRI probes utilizing nanotechnology holds significant potential for clinical translation.

### 6.3. Multimodal Imaging Innovations

**Figure 8:**
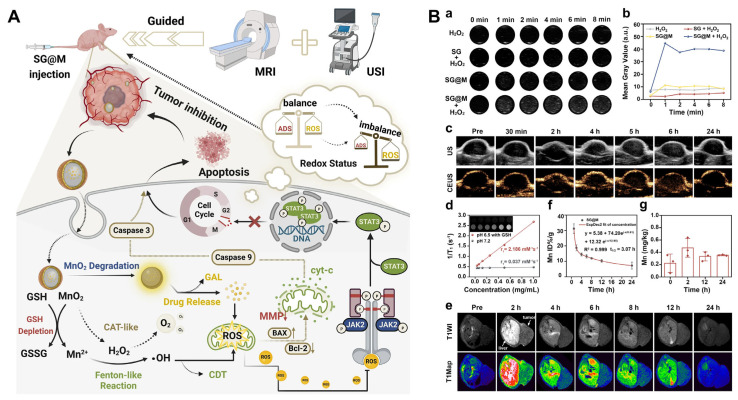
Multimodal imaging-guided therapy for pancreatic cancer. (**A**) Schematic representation of the preparation process and mechanism of action for SiO_2_-GAL@MnO_2_ (SG@M). (**B**) MRI and ultrasound imaging effects of SG@M. (**a**) In vitro ultrasound images of different samples at various time points; (**b**) Corresponding mean gray values; (**c**) In vivo ultrasound and contrast-enhanced ultrasound images of tumors before and after intravenous injection of SG@M at different time points (Scale bar not present in original image); (**d**) T_1_-weighted MR images of SG@M at varying concentrations in pH 7.2 and pH 6.5 buffers containing GSH (5 mM), along with relative T_1_ relaxation rates; (**e**) T_1_-weighted MR images of tumor-bearing mice before and after intravenous injection of SG@M at different time points (Scale bar not present in original image); (**f**) Blood circulation profile of Mn in tumor-bearing mice administered SG@M (n = 3); (**g**) Accumulation of Mn in tumor tissues (n = 3). Copyright 2023, with permission from Springer Nature [208].

Multifunctional nanoplatforms that integrate multimodal imaging contrast agents and therapeutic agents have garnered significant attention owing to their capability to surmount the limitations inherent in single-mode imaging. Researchers have developed a novel hybrid nanomedical agent to enhance the therapeutic efficacy of pancreatic cancer treatment by amplifying oxidative stress and enabling dual-mode imaging via ultrasound and MRI (Figure 8A). This nanomedical formulation comprises silica nanoparticles loaded with galangin as the core, encapsulated within an MnO_2_ layer, forming SiO_2_-GAL@MnO_2_ nanospheres. In the tumor microenvironment, the MnO_2_ layer reacts with and depletes GSH, generating Mn^2+^, which catalyzes Fenton-like reactions, producing ·OH that induces ferroptosis. Additionally, Mn^2+^ facilitates T1-weighted MRI imaging. The catalase-like activity of MnO_2_ also enables the generation of O_2_ for ultrasound imaging applications (Figure 8B). Upon degradation of the MnO_2_ shell, galangin is released from the biodegradable SiO_2_ carrier, stimulating ROS production and further enhancing oxidative stress-induced damage in tumor cells. Excessive ROS induces oxidative stress-mediated mitochondrial dysfunction and a reduction in mitochondrial membrane potential. This damage prompts the release of cytochrome c, which subsequently activates the Caspase 9/Caspase 3 apoptotic cascade pathway. Concurrently, ROS-induced decreases in JAK2 and STAT3 phosphorylation inhibit the JAK2/STAT3 cell proliferation pathway, leading to reduced Cyclin B1 protein levels and cell cycle arrest in the G2/M phase. The synergistic effects of the Fenton reaction and apoptosis strongly suppress pancreatic cancer progression, with a tumor growth inhibition rate of 62.7%. Overall, this study demonstrates that hybrid nanomaterials offer advantages in augmenting tumor oxidative stress, providing a novel strategy for pancreatic cancer treatment through a catalytic cascade effect. Moreover, dual-mode imaging guidance offers critical support for precise drug localization and monitoring [208]. Therefore, multimodal image-guided therapy integrates various imaging modalities to offer more comprehensive and precise information regarding the lesion, thereby assisting researchers in devising more personalized and targeted treatment strategies.

## 7. Future Directions and Challenges

While the combination of ferroptosis, nanotechnology, and image-guided therapy offers promising new avenues for the treatment of pancreatic cancer, several challenges must be addressed before they can be widely used in the clinic. Regardless of its application prospects in cancer treatment, ferroptosis still awaits prioritization in both preclinical and clinical settings. The primary therapeutic obstacle of ferroptosis lies in tumor heterogeneity. Due to variations in cellular iron levels and gene expression associated with ferroptosis, different individuals may respond differently to this treatment approach. Therefore, iron levels, gene expression, and mutations can be utilized to identify patient populations that are likely to respond to ferroptosis-inducing drugs. Interestingly, p53 plays a critical role in the regulation of ferroptosis. Xie et al. found that the tumor suppressor *TP53* blocks the activity of dipeptidyl peptidase-4 (DPP4) in a transcription-independent manner, rendering cells resistant to erastin and thereby inhibiting ferroptosis [209]. Another study revealed that high levels of GPX4 do not mediate ferroptosis. Knockout of GPX4 leads to elevated intracellular iron levels, ultimately resulting in lipid peroxidation and ferroptosis. This demonstrates that ferroptosis is attenuated under conditions of GPX4 overexpression [210]. Another mechanism controlling ferroptosis, independent of the GPX4/glutathione system, is the GCH1/BH4 pathway. The production of tetrahydrobiopterin (BH4) is slowed by the enzyme GTP cyclohydrolase 1 (GCH1). Research has shown that overexpression of GCH1 can eliminate lipid peroxidation and almost entirely suppress ferroptosis [211]. From a pharmacokinetic perspective, traditional ferroptosis inducers face challenges, such as poor water solubility, limited bioavailability, and drug resistance. Erastin serves as a prime example, exhibiting unstable metabolism and poor water solubility, leading to significant side effects [212]. Another major challenge is the in vivo application of ferroptosis inducers. For instance, drugs such as sulfasalazine, sorafenib, cisplatin, and artemisinin are currently considered to be promising ferroptosis inducers; however, severe adverse drug reactions, non-specific distribution, and high dosage requirements limit their clinical application [213]. Therapeutic strategies should be designed to selectively target specific cancer cells without affecting normal or healthy cells. The failure of ferroptosis inducers, apoptosis inducers, or their combinations to reach intended or desired locations disrupts healthy cells, thereby reducing median survival rates due to multi-organ failure or multidrug resistance. Thus, nanotechnology offers promising advantages in overcoming these barriers.

The dense fibrotic matrix of pancreatic cancer is one of the key factors hindering the success of treatment. Not only does the dense matrix hinder the delivery of therapeutic drugs, but CAFs in the matrix can also inhibit ferroptosis by secreting cytokines. While stroma elimination was envisioned as revolutionizing the treatment of pancreatic cancer, some clinical trials targeting stroma have brought disappointing results, such as the inhibition of profibrogenic pathways using the sonic hedgehog (SHH) pathway inhibitor [214,215,216] and the degradation of the extracellular matrix using PEGylated hyaluronidase (PEGPH20) [217,218]. However, the focus of research has now shifted to matrix regulation rather than consumption. Nanomedicine provides some answers, such as nanomaterial-loaded SHH inhibitors that provide matrix regulation [219]. In addition, metformin and the angiotensin II receptor antagonist Losartan have shown promise in matrix remodeling and have been shown to enhance the delivery and efficacy of chemotherapy agents [220,221,222]. In fact, some anti-stromal therapies have demonstrated that alterations in the tumor immune landscape, such as altered macrophage polarization, can make tumor cells sensitive to ferroptosis [147,148]. Therefore, combining nanotechnology with matrix regulators could potentially improve intratumoral delivery of ferroptosis inducers.

Another characteristic of pancreatic cancer is its highly immunosuppressive tumor microenvironment. Immunosuppression is mainly manifested in the immune surveillance evasion of tumor cells, the participation of a variety of immunosuppressive cells, and the expression of a variety of immunosuppressive molecules. Pancreatic cancer cells directly inhibit T cell activity through high expression of PD-L1, TIM-3 and other immune checkpoint molecules, thereby escaping immune attack. Immunosuppressive cells, such as bone marrow-derived suppressor cells (MDSC), infiltrate in large numbers in pancreatic cancer, inhibit T cell function by secreting arginase and ROS, and promote the expansion of regulatory T cells (Tregs), further exacerbating immunosuppression. CAFs form a physical barrier by secreting the extracellular matrix, resulting in increased interstitial pressure and hypoperfusion. The associated hypoperfusion creates a hypoxic microenvironment that results in Treg-mediated CD8^+^ T cell inhibition [223,224]. Beyond the physical barrier, a recent study found that autophagy in CAFs promotes the “target loss” of CD274 through the IL6/USP14/CD274 signaling pathway, further contributing to the formation of an “immune desert” in pancreatic cancer, thereby enabling evasion from CD8^+^ T cell-mediated killing [225]. Owing to the presence of multiple immunosuppressive mechanisms, existing immunotherapy strategies yield limited efficacy in the clinical management of pancreatic cancer. Therefore, an increasing number of studies are focusing on exploring novel combination strategies for immunotherapy. Ferroptosis has been shown to increase cellular immunogenicity through a variety of pathways. Mechanically, damage to cell membrane structure during ferroptosis can lead to the release of endogenous damage-associated molecular patterns (DAMPs), which are recognized by antigen-presenting cells (APCs) to induce an enhanced innate immune response [226]. In addition, multiple regulators of the ferroptosis pathway have been shown to make cancer cells sensitive to immunotherapy. For example, when used in combination with GPX4 inhibitors, cancer cells and xenograft tumors increase sensitivity to the chemotherapy drug gemcitabine by inducing ferroptosis [227]. Therefore, the immune-ferroptosis nanovaccine therapeutic strategy may be a promising therapeutic strategy to improve the immune deficiency status of pancreatic cancer. For example, nanocarriers designed to encapsulate ferroptosis inducers and neoantigens promote APC cell activation and CD8^+^ T cell response, thereby enhancing pancreatic cancer immunotherapy efficacy. Further, imaging can be used to monitor the degree of immune response and the infiltration of immune cells, to achieve accurate and effective immune-ferroptosis combination therapy.

The safety issue of nanotechnology-based ferroptosis is another key issue, such as the excessive accumulation of iron ions that may lead to toxic reactions. To address the safety concerns caused by metal ion release, multiple optimization strategies can be intelligently integrated to balance the risks. Specifically, biodegradable carriers, such as metal–organic frameworks (MOFs), can enhance biocompatibility [228]. Surface PEGylation or biomimetic cell membrane coating can effectively reduce immune clearance [229]. Stimuli-responsive release systems, such as light/ROS-triggered mechanisms, enable precise control over ion release [230,231]. By integrating these strategies into nanoparticle design, the potential toxicity risks can be transformed into therapeutic benefits. Furthermore, the results of in vivo efficacy and safety studies conducted in animal models do not necessarily reflect equivalent results in humans, as the in vivo fate of nanomaterials and their interactions with blood components can be highly variable [232,233]. While nanoparticles have shown promising targeting capabilities, their biocompatibility, potential immune responses, and long-term effects are still not fully understood [234]. Further studies are necessary to fully evaluate the safety of nanoparticles and to avoid causing adverse reactions or chronic toxicity. Artificial intelligence (AI) is transforming the field of nanomedicine by exploring new nanomaterials to develop highly effective treatments. AI, which relies on large models and big data to work, is able to find suitable nano properties for different therapeutic targets, and is expected to eventually improve the safety and effectiveness of nanomaterials [235]. AI uses patients’ clinical and genetic data to predict outcomes and guide treatment, improving efficacy while minimizing side effects [235]. AI can achieve personalized prediction of nanoparticle biological distribution based on tumor matrix density and metabolic profile characteristics through machine learning models [236,237]. Consequently, AI-driven nanomedicine is anticipated to bring about revolutionary advancements in the next generation of nanotechnology.

Finally, the clinical translation of these technologies still faces some obstacles, including the need for rigorous clinical validation, the technical feasibility of implementing such complex therapies, and the significant financial investment required for large-scale production of nanomaterials. Although preclinical studies have shown promising results, translating these advanced therapies into effective clinical treatment options requires extensive clinical trials and regulatory approval. Despite the challenges, advances in technology and research show that this cutting-edge technology has great potential to improve the treatment of pancreatic cancer. As technology continues to evolve, multidisciplinary integrated treatment strategies that combine ferroptosis, nanotechnology, and image-guided therapy are expected to play a key role in the treatment of pancreatic cancer, thereby improving the survival prospects of patients.
